# The thorax morphology of *Epiophlebia* (Insecta: Odonata) nymphs – including remarks on ontogenesis and evolution

**DOI:** 10.1038/srep12835

**Published:** 2015-08-06

**Authors:** Sebastian Büsse, Benjamin Helmker, Thomas Hörnschemeyer

**Affiliations:** 1 University Museum of Zoology, Department of Zoology, University of Cambridge, Downing Street, CB2 3EJ Cambridge, UK; 2 J.- F.- Blumenbach Institute for Zoology & Anthropology, Department Morphology, Systematics & Evolutionary Biology Georg-August-University Göttingen, Berliner Str. 28, 37073 Göttingen, Germany

## Abstract

The species of *Epiophlebia* are unique among the recent Odonata in showing a mixture of morphological characters of dragonflies (Anisoptera) and damselflies (Zygoptera). The status of the four described extant species of *Epiophlebia* is disputable from a genetic as well as from a morphological point of view. Here we present an analysis of the thoracic musculature of different nymphal instars of *Epiophlebia laidlawi* and *Epiophlebia superstes* to elucidate their morphology and ontogenetic development. In total, 75 muscles have been identified in the thorax of *Epiophlebia*. This represents the highest number of thoracic muscles ever found in any odonate. It includes six muscles that are reported for the first time for Odonata, and three of these are even new for Pterygota. In total, our results indicate that *Epiophlebia* has the most ancestral thoracic morphology among Odonata.

Almost all known recent Odonata can be assigned to one of two groups: either to the Anisoptera (dragonflies) or to the Zygoptera (damselflies). A conspicuous exception are the few species of *Epiophlebia*, which combine characteristics of both groups^1^,^2^. *Epiophlebia* nymphs resemble those of the Anisoptera, respiring through a rectal chamber, while lacking the paddle shaped gills that arise from the tip of the abdomen^1^ and are characteristic of Zygoptera. Jet propulsion, an otherwise common behaviour in dragonfly nymphs, has not been documented for *Epiophlebia*^3^. At first glance the body-shape of *Epiophlebia* adults resembles the anisopteran type. Closer examination shows, that its fore- and hind wings look similar, are stalked and held together above the abdomen when in resting position, which is quite similar to what can be found in Zygoptera. Based on this presumably ancestral^4^ mixture of characters, *Epiophlebia* has been called a “living fossil”^1^, a relic, which was supposed to be the last extant member of a taxon that otherwise comprised mainly Jurassic species, the “Anisozygoptera”^5^. The “Anisozygoptera” were shown to be paraphyletic by Nel *et al.*^5^ and Lohmann^6^ later suggested a sister-group relationship between *Epiophlebia* and Anisoptera. This grouping was named Epiprocta with *Epiophlebia* on the most basal split in the Epiprocta-tree, followed by a comb of several extinct taxa on the branch leading to Anisoptera^6^.

## Biogeography of ***Epiophlebia***

Apart from its peculiar morphology, *Epiophlebia* also puzzled odonatologists by its distribution. The first species described, *Epiophlebia superstes* Sélys, 1889^7^, is a common insect in Japan, whereas *Epiophlebia laidlawi* Tillyard, 1921^8^ was discovered in small mountain enclaves in the Himalayas of India, Nepal^9^,^10^ and Bhutan^11^. It took 90 years to finally reduce this 5000 km gap by spotting a third species, *Epiophlebia sinensis* Li & Nel, 2011^12^, in Northeast China and a fourth, *Epiophlebia diana* Carle, 2012^13^, in Central-China. The latter three species have been poorly documented so far. Especially in the cases of *E. sinensis* and *E. diana* this is due to the very low number of collected specimens (two male adults of *E. sinensis*, two nymphs of *E. diana*). *E. laidlawi* lives in the Himalayas in isolated subtropical pine-forests^9^ in altitudes up to 3600 m a.s.l.^14^. According to Davies^15^, *E. laidlawi* flies on sunny mountaintops, which emerge above the cloud cover, and it breeds close to high waterfalls. The nymphs favour fast running mountain streams at altitudes between 2200 to 2700 m a.s.l.^14^, with temperatures ranging from 3.1 to 17.9 °C throughout the year and water currents reaching 200 cm/s^11^. Tabaru^3^ reported that the nymphs of *E. superstes* undergo fourteen instars over a period of five to nine years until the last instar finally emerges from the water in spring.

Büsse *et al.*^2^ investigated phylogeographic aspects of the isolated *Epiophlebia*-populations. It was revealed that the degrees of similarity between the sequences of sections of 18S & 28S rDNA, ITS1, ITS2 and CO2 of *E. superstes*, *E. laidlawi* and *E. sinensis* are remarkably high. The genetic differences between the three species resemble those otherwise found between different populations of the same species in Odonata^2^. Furthermore, the validity of the newly described species of *Epiophlebia* – *E. sinensis* and *E. diana* – is challenged by odonatologists^16^.

### Insect flight and thorax morphology

The evolutionary success of pterygote insects can be attributed largely to the evolution of the ability to fly^17^. Despite of this evolutionary importance, the origin and evolutionary development of the insect flight apparatus are still only partially understood^17^,^18^,^19^,^20^,^21^. Within Pterygota, the Odonata are among those groups that show the most impressive flight skills^22^.

The mechanism of wing movement is realized in different ways among Pterygota: The Odonata have an exclusively direct flight mechanism where dorso-ventral muscles are attached to elements of the wing base, actuating the wings directly. Dorsal longitudinal muscles, which are a crucial part of the flight musculature of all other pterygote insects, are either extremely small or missing in Odonata^18^,^23^,^24^,^25^. The mechanism that drives the wings in all other Pterygota works largely indirectly through deformation of the winged thoracic segments while the usually weaker direct flight muscles are mainly responsible for steering and flight control actions, e.g. adjusting the wing’s angle of attack, etc.

As exclusively aerial predators adult Odonata depend even more on the performance of their flight apparatus than many other Pterygota^23^. Therefore, understanding its complex morphology is necessary for a better understanding of behaviour, phylogeny and evolution of this group.

The thoracic musculature of adult Epiprocta^1^,^23^,^24^,^25^ as well as the pterothorax^18^, and to some degree the entire thorax^1^,^19^ of adult Zygoptera have been comprehensively investigated. The morphology and development of the nymphal thorax of Odonata, however, have only been studied superficially^1^,^20^,^26^,^27^. The present investigation of the ontogenesis of the thoracic musculature of *Epiophlebia* nymphs will substantially supplement the hitherto available information, leading to a better understanding of the evolution and development of the odonate thorax.

## Results

To compare the thoracic muscles of *E. laidlawi* and *E. superstes* with Anisoptera, Zygoptera as well as Neoptera, each muscle is identified according to the homologization proposed by Büsse *et al.*^18^ and the muscle nomenclature proposed by Friedrich & Beutel^28^.

In total 75 muscles are identified in the thorax of *Epiophlebia* nymphs, 20 in the prothorax, 26 in the mesothorax and 29 in the metathorax (Table 1). This represents the highest number of thoracic muscles ever found in a species of Odonata^1^,^18^,^20^,^27^.

Detailed descriptions of the muscles together with information on the interpretation of their identity or homology are given in Supplementary data 1.

Cuticle (Supplementary Figures 1–3). 

For the skeletal elements of the thorax we use the nomenclature of Asahina^1^. Where necessary, this is supplemented with terms from Snodgrass^29^ and Ninomiya & Yoshizawa^19^.

The cuticle of *Epiophlebia* nymphs is ca. 1.5 to 2 times thicker than that of other Odonata investigated. The smooth sternites (Supplementary Figures 1& 2) each display two prominent furcal pits. On the inside, the corresponding three pairs of cone-shaped furcae (Supplementary Figures 1& 2: pro- (F1), meso- (F2) and metafurca (F3)) are attachment points for several muscles (see Supplementary data 1).

On the postero-lateral surface of each coxa a short process is present, which serves as attachment point for the large pronator muscle of each leg.

The tergites of the three thoracic segments (Supplementary Figure 3) are substantially different from each other. The prothoracic tergite is a broad plate covering the entire dorsum of the prothorax. In the centre of the prothoracic tergite a small spur, the first tergal apophysis, is present (Supplementary Figure 3: TEa1). The second, third and fourth tergal apophyses are represented by the segmental borders between pro- and mesothorax, meso- and metathorax and mesothorax and first abdominal segment, respectively (Supplementary Figure 3: TEa2, TEa3, TEa4). In the cranial half of the dorsal part of the mesothorax both pleurites arch towards each other and displace the mesothoracic tergite to a more caudal position. The meso- and metathoracic tergites are covered by the wing buds and each bears a stubby lateral process, which serves as attachment point for muscles (see Supplementary data 1).

The pleurites (Supplementary Figure 1) are divided into the episternum and the epimeron. Whereas the prothoracic pleurite has no distinct apodemes or extensions, the meso- and metathoracic pleurites have prominent arched interpleural ridges. The metathoracic preepisternal apodemes arise just behind the intersegmental border. The spoon-shaped structures on both sides of the body extend towards the median axis and are connected by a transverse muscle (see Supplementary data 1), above the nervous system. Asahina^1^ described a mesothoracic preepisternal apodeme in *Epiophlebia superstes* that serves as an attachment point for transverse muscles. Although these muscles could be identified in both species of *Epiophlebia*, determining the exact outline of the mesothoracic preepisternal apodemes was not possible in the specimens examined.

## Discussion

In the following section the nymphal musculature of *E. laidlawi* and *E. superstes* is compared with that of zygopteran and anisopteran nymphs. Additional information is taken from the descriptions of Maloeuf^27^ and Büsse & Hörnschemeyer^20^ for Anisoptera, from Asahina’s^1^ work on *E. superstes* and from the analysis of the musculature of adult Zygoptera by Büsse *et al.*^18^. A comparison of muscle nomenclatures of different authors can be found in Supplementary table 1.

The muscle numbers used by Maloeuf^27^ and Asahina^1^ are given in parenthesis. An additional number in parentheses within the first set denotes the homologous muscle in the meso- or metathorax. Muscles not recognized by Maloeuf^27^ or Asahina^1^ are marked with (-). Muscles not mentioned by Friedrich & Beutel^28^ are marked with * and named according to their points of origin and insertion. Their abbreviations are numbered consecutively (Table 1, Supplementary data 1).

### Dorsal longitudinal muscles

**Figure 1:**
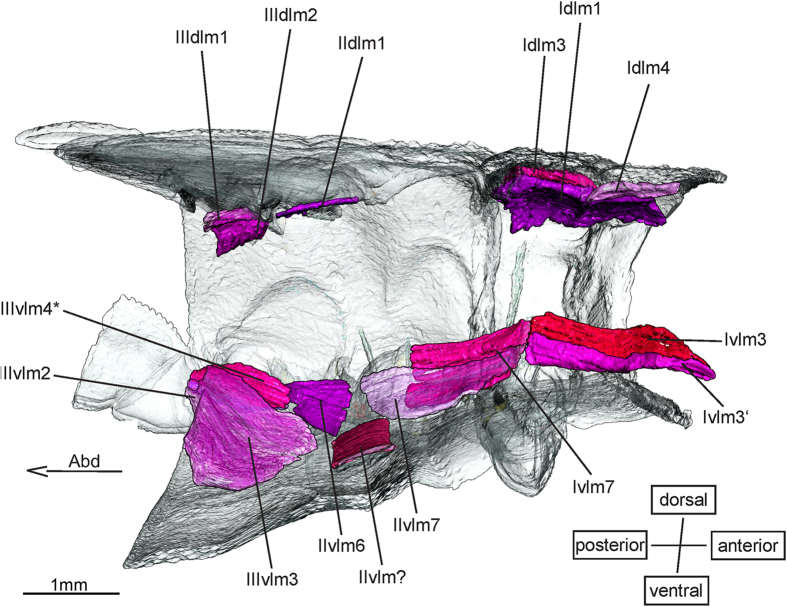
Dorsal longitudinal and ventral longitudinal musculature of *Epiophlebia laidlawi*. 3D - reconstruction from SRμCT data showing the left half of the thorax. Abd - Abdomen, dlm - dorsal longitudinal muscle, vlm - ventral longitudinal muscle.

The dorsal longitudinal muscle **IIdlm1** (Fig. 1) is small and is missing in nymphal Zygoptera. However, it is present but very small, consisting of just a few fibres, in adult Zygoptera^18^. This might indicate that IIdlm1 develops only in the latest instars and was not present in the instars investigated. It is present in nymphs of Anisoptera^20^.

### Dorsoventral muscles

**Figure 2:**
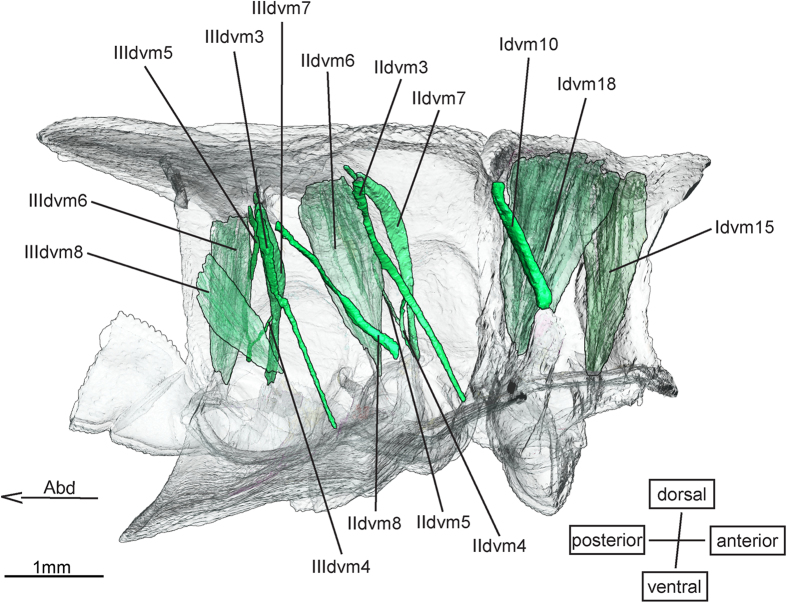
Dorso-ventral musculature of *Epiophlebia laidlawi*. 3D - reconstruction from SRμCT date showing the left half of the thorax. Abd - Abdomen, dvm - dorso-ventral muscle.

**Idvm18** (14) (Fig. 2) originates from a large area of the prothoracic tergum that also encompasses the origin of Itpm3. Idvm18 inserts on an apodeme on the posterior base of the procoxa. It is by far the largest prothoracic muscle. Maloeuf^27^ described its lateral branch as a discrete muscle (15). Idvm18 does show a slight dichotomy, yet all of its fibres run from the tergum to the apodeme. *E. superstes* and *E. laidlawi* show the same characteristics. Neither in Anisoptera nor in Zygoptera Idvm18 shows any striking dichotomy that would suggest the presence of an independent muscle (15)^18^,^20^.

A muscle homologous to **II (III)dvm7** (-) is not directly described by Maloeuf^27^ nor Asahina^1^, yet in Maloeuf’s table 8 muscle IIdvm4 is listed as being dichotomous. Given the position of IIdvm4, it is quite probable that one of its alleged portions in fact is equivalent to muscle IIdvm7. In the nymphs of Anisoptera^20^ and in the adults^18^ and nymphs of Zygoptera muscle II (III)dvm7 is missing. In the nymphs of *E. laidlawi* and *E. superstes* it originates anterior-median to II (III)dvm4, runs parallel to it and finally is attached through a tendon to the trochanter while II (III)dvm4 inserts laterally on the coxa. Consequently, both muscles II (III)dvm4 as well as II (III)dvm7 seem to be present in nymphs and in adults of *Epiophlebia*.

In Neoptera muscle II (III)dvm7 originates at the central region of the notum of its segment and inserts at the trochanter^28^. In the *Epiophlebia* nymphs II (III)dvm7 originates at the antero-ventral rim of the meso- and metathoracic wing bud, which is a part of the notum.

**IIdvm8** (-) is an intersegmental muscle, stretching between the mesofurca and the metathoracic tergite. Its homologues are Idvm10 and IIIdvm8 as they connect similar structures. Muscle IIdvm8 was not found in Anisoptera^20^, or in the zygopteran thorax^18^ but in Neoptera^28^ and in both species of *Epiophlebia*. Therefore, IIdvm8 probably is a muscle of the pterygote ground pattern that was, among the Odonata, only preserved in *Epiophlebia*.

The four muscles **II (III)dvm1** and **II (III)dvm2** are not present in *Epiophlebia* but could be confirmed for Zygoptera nymphs. Muscle II (III)dvm1 is present in nymphs of Anisoptera and in adult Zygoptera^18^,^20^.

Muscle **II (III)dvm3** seems to be unique for Zygoptera nymphs^18^,^20^. The muscles **II (III)dvm2** were found in the nymphs of Zygoptera for the first time. They show the same points of origin as in Neoptera^28^, whereas the insertions lie at the anterior margins of the corresponding coxae and not on the trochantins as described by Friedrich and Beutel^28^. However, free trochantins are not present in Odonata^30^ and the points of insertion of II (III)dvm2 on the coxae may well represent the positions where the trochantins are fused to the coxae.

### Sterno-coxal and pleuro-coxal muscles

**Figure 3:**
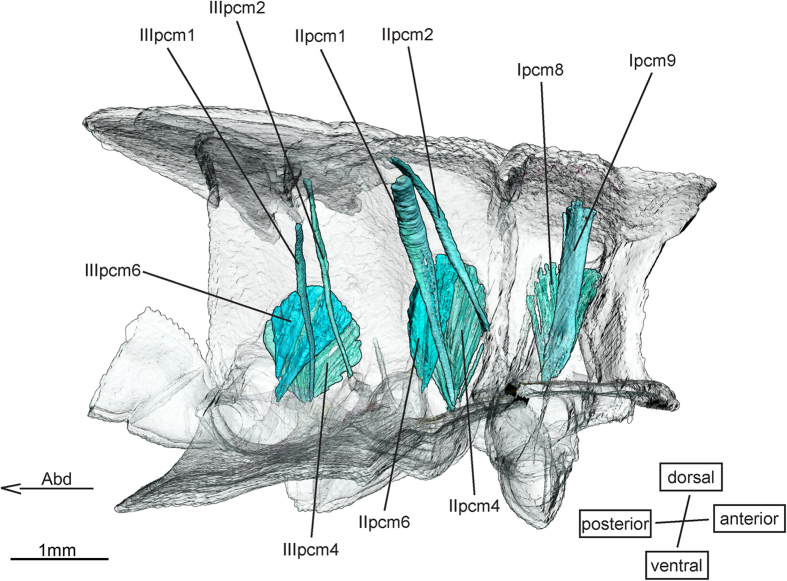
Pleuro-coxal musculature of *Epiophlebia laidlawi*. 3D - reconstruction from SRμCT data showing the left half of the thorax. Abd - Abdomen, pcm - pleuro-coxal muscle.

The pleuro-coxal muscles (Fig. 3) **II (III)pcm2** are among those that undergo the most extensive growth in the pterothoracic segments. They start out very slender in the early instars and grow to be among the largest muscles in the respective segment in the latest instars. In contrast to the description by Asahina^1^, we found clearly separated origins of II (III)pcm2 and II (III)pcm1.

**Figure 4:**
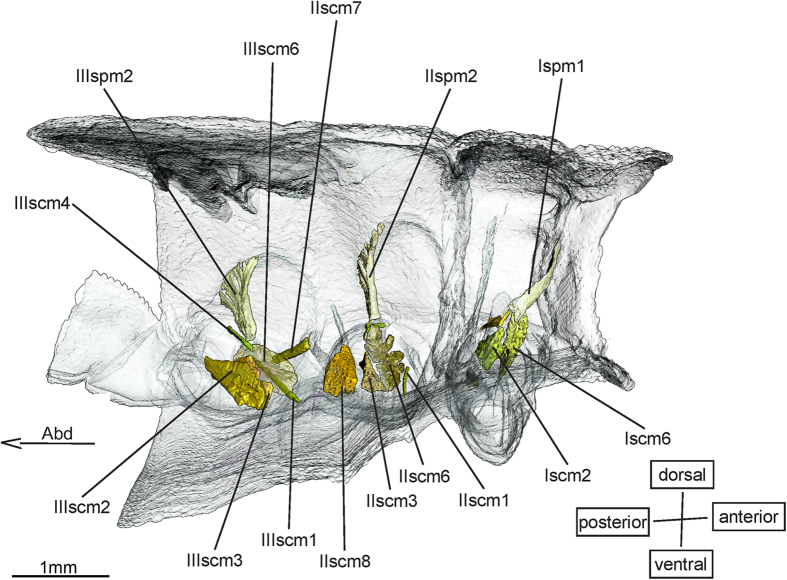
Sterno-coxal and sterno-pleural musculature of *Epiophlebia laidlawi*. 3D - reconstruction from SRμCT data showing the left half of the thorax. Abd - Abdomen, scm - sterno-coxal muscle, spm - sterno-pleural muscle.

Among the sterno-coxal muscles (Fig. 4) **Iscm4** (-), **IIscm4** (-) & **IIIscm4** (-) were not mentioned by Maloeuf^27^ or Asahina^1^. All three muscles were found in the nymphs of *E. laidlawi* and *E. superstes.* A possible explanation could be that all three muscles only exist in juvenile stages. At least IIIscm4 is also present in nymphs of Anisoptera^20^ and IIscm4 was found in Zygoptera nymphs. However, none of the three muscles have been found in adult Zygoptera^18^. The homology established for IIscm4 in Büsse & Hörnschemeyer^20^ holds for Iscm4 and IIIscm4 as well.

**IIscm1** (-) & **IIIscm1** (-) are very thin muscles running close to the meso- and metasternum and inserting by means of an apodeme on the lateral side of the coxa. These two muscles were neither listed by Maloeuf^27^ nor Asahina^1^, but are present in *E. laidlawi*, *E. superstes*, in the Zygoptera and Anisoptera^20^. Not being mentioned by Büsse *et al.*^18^ for adult Zygoptera, IIscm1 and IIIscm1 seem to be exclusively nymphal muscles in Odonata.

**IIscm8** (-) is a funnel shaped muscle connecting the mesothoracic coxa and the metathoracic preepisternal apodeme. It has no serial homologue in the pro- or metathorax. Its presence is confirmed for both species of *Epiophlebia,* the Zygoptera and the anisopteran nymphs^20^. Neither Maloeuf^27^ nor Asahina^1^ or Büsse *et al.*^18^ mentioned IIscm8, indicating that it also is a muscle that is restricted to Odonata nymphs.

**IIIscm2** (-) connects the base of the metafurca and the posterior base of the metacoxa. It is confirmed for *E. laidlawi*, *E. superstes*, the Zygoptera and the Anisoptera^20^. Since it was not found in investigations of adult Odonata^1^,^18^,^27^, it probably also is an exclusively nymphal muscle. In the nymphs of the Ansioptera the homologue muscle IIscm2 could be identified^20^.

### Sterno-pleural muscles

The sterno-pleural muscle (Fig. 4) **Ispm1** (-) originates from the lateral surface of the apex of the profurca and inserts in the anterolateral area of the prothoracic epimeron. It was found in *E. laidlawi, E. superstes,* in Zygoptera and in Anisoptera nymphs^20^. Ispm1 resembles IIspm2 and IIIspm2, which originate from the apex of the meso- and metafurca, but it inserts in a different area. Ispm1 is a nymphal muscle^1^,^18^,^27^.

### Tergo-pleural muscles

**Figure 5:**
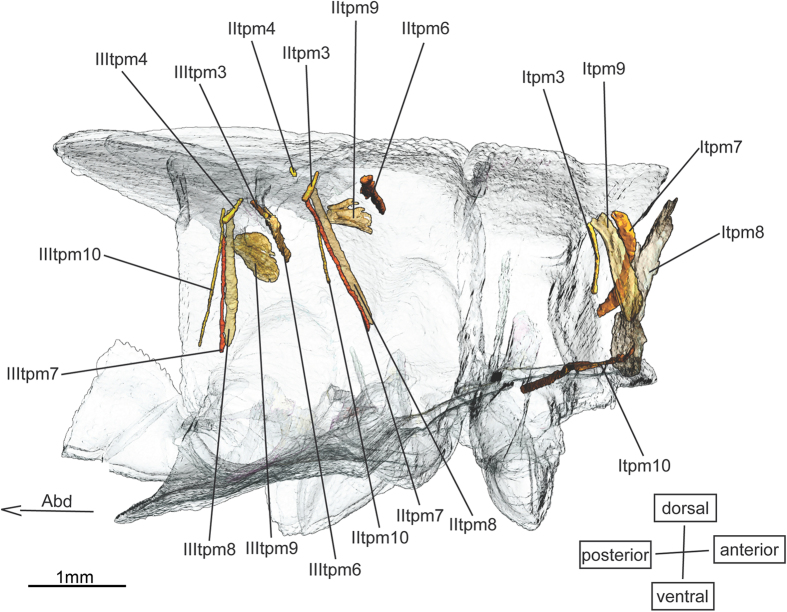
Tergo-pleural musculature of *Epiophlebia laidlawi*. 3D - reconstruction from SRμCT showing the left half of the thorax. Abd - Abdomen, tpm - tergo-pleural muscle.

The origins and the insertions of the tergo-pleural muscles (Fig. 5) **IItpm3** (-) & **IIItpm3** (-) lie inside the wing buds. They are the smallest muscles in the nymphs. Both were found in the nymph of the two species of *Epiophlebia* and in the nymphs of the Anisoptera^20^. They are not present in the nymphs of the Zygoptera and are not known for adults of Zygoptera^18^ or Epiprocta^1^,^27^. Probably, they only occur in juvenile Epiprocta.

Similarly, the muscles **Itpm7**, **Itpm8** and **Itpm9** are found exclusively in Epiprocta nymphs.

### Transverse muscles

**Figure 6:**
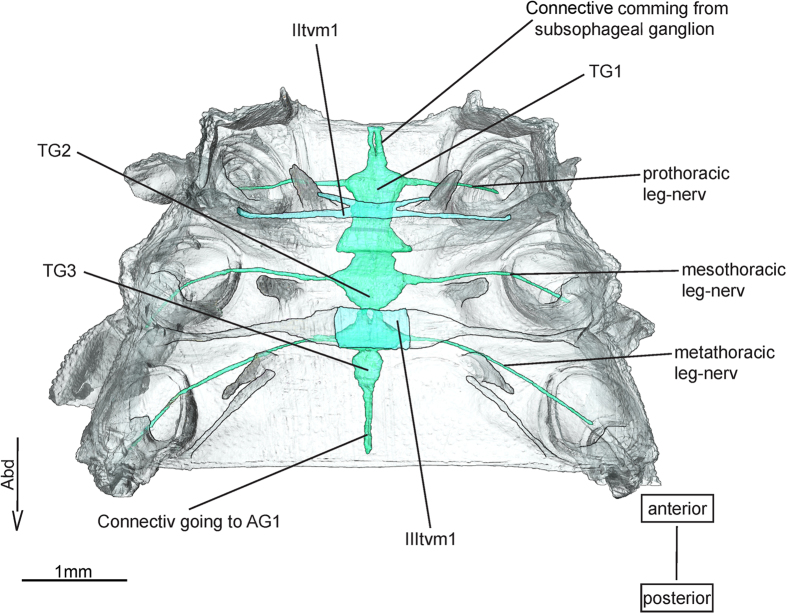
Transverso-ventral musculature and ventral nerve cord of *Epiophlebia laidlawi*. 3D - reconstruction from SRμCT data showing the ventral half of the thorax. AG - abdominal gaglion, Abd - Abdomen, TG - thoracal ganglion, tvm - transverso-ventralmuscle.

The transverse ventral muscle (Fig. 6) II (III)tvm1* (-) was described by Maleouf^27^ as muscle (69). It is supposed to run transversely between intersegmental sterno-pleural processes. These processes, depicted by Maloeuf^27^ in an adult dragonfly, have been named preepisternal apodemes by Asahina^1^, who also found IItvm1 in the nymphs of *E. superstes*. However, both authors never described a transverse muscle by name. Maki^30^ indicates the presence of a transverse muscle but gives no description. This muscle is neither present in Zygoptera nor in Anisoptera^18^,^20^.

### Ventral longitudinal muscles

Among the ventral longitudinal muscles (Fig. 1) **Ivlm3** (11) & **Ivlm3’** (11’) have been found in adults of Epiprocta^1^,^27^. *E. laidlawi*, *E. superstes* and the nymphs of the Anisoptera^20^ do not show a division of Ivlm3. It is also not reported for Neoptera^28^. The only argument for identifying Ivlm3’ as a separate unit is the fact that it does not insert directly on the tentorial bar, like Ivlm3, but on a membrane right underneath it. It is very likely that Ivlm3’ is only a separate strand of Ivlm3, which is missing in Zygoptera^18^. Muscle IIvlm3 is only present in zygopteran nymphs and IIIvlm3 is only present in Epiprocta^20^.

**IIvlm7** (42) is a longitudinal muscle connecting the profurca and the metathoracic preepisternal apodeme. According to Asahina^1^, it supposedly connects the profurca and the first abdominal segment through IIvlm6 (68). Büsse & Hörnschemeyer^20^ stated that IIvlm7 connects the metafurca and the abdomen in Anisoptera, an interpretation confirmed here. In *E. laidlawi* and *E. superstes*, however, IIvlm7 does not continue through the metathorax. It inserts on the anterior margin of the metathoracic preepisternal apodeme. Muscle IIvlm7 is missing in the Zygoptera nymphs investigated.

**IIvlm6** (68) originates from the posterior surface of the metathoracic preepisternal apodeme. It has exactly the same width as IIvlm7 and inserts on the anterior process of a thin structure arising from the ventral phragma of the first abdominal segment. This structure might either be a cap tendon or a very fragile apodeme, since it could not be properly identified from the datasets. It might be a nymphal muscle since it is present in immatures of Zygoptera and Anisoptera^20^, but not in adult Zygoptera^17^. Muscle Ivlm6 only occurs in Zygoptera nymphs.

IIIvlm4* (64) has its origin on the anterior process of the thoracic-abdominal tendon mentioned above, just posterior to IIvlm6. It is the last muscle in a row of longitudinal muscles connecting the tentorial bar, the furcae and the abdomen. It is missing in nymphs of Anisoptera^20^, but is present in all examined nymphs of *Epiophlebia*. In adults of Zygoptera IIIvlm4 is replaced by a tendon, which connects the metafurca with the bar between the first and second abdominal segment^17^. Muscle IIIvlm4 is missing in nymphs of Zygoptera. According to Maloeuf^27^ this muscle is absent in adult Odonata, but Asahina^1^ identified it in adults of *E. superstes*. A possible explanation is that IIIvlm4 retracts at a certain stage into the abdomen, which gets elongated after the last ecdysis. Further examination might be necessary, to clarify the status of IIIvlm4.

Muscles **Ivlm6** und **IIvlm3** could be identified in nymphs of Zygoptera, the first evidence for the presence of theses muscles in Odonata. Muscles Ivlm6 and IIvlm3 show exactly the same attachment points as described for Neoptera^28^.

### Muscle summary

All muscles described for the Odonata by Maloeuf^27^ and for adults of *E. superstes* by Asahina^1^ could be identified in the nymphs of *E. superstes* and *E. laidlawi*. Five muscles differ from the descriptions of both authors: Ivlm3, muscle, IIvlm7, IIvlm6 and IIIvlm4. Six muscles, IIIvlm4, II (III)tvm1, Iscm4, IIdvm7 and IIIdvm7 could be newly identified in the Odonata^1^,^18^,^20^,^27^.

In Anisoptera four muscles are present that are missing in *Epiophlebia*: II (III)dvm1, IIscm2 and IIscm7.

The Zygoptera have eight muscles, which are not present in *Epiophlebia*: II (III)dvm1, II (III)dvm2, II (III)tpm2, Ivlm6 and IIvlm3.

These results confirm that *E. superstes* and *E. laidlawi* are highly similar in almost all aspects of their thoracic morphology as well as on the genetic level, as stated by Büsse *et al.*^2^.

Poletaïev^31^ reported that the wing buds of Odonata appear in the 3^rd^ or 4^th^ instar but that the corresponding musculature is still indiscernible. Maloeuf^27^ noted that the flight muscles in these instars are still diminutive. Our investigation confirms Maloeuf’s observation: some flight muscles of the adult first appear in early instars as sets of very few muscle fibres. Some are scarcely traceable, like for instance Itpm10 and Itpm11, and then grow significantly during ontogenesis, like II (III)dvm3, II (III)pcm2, II (III)tpm7.

Some other muscles, like IIIdlm1, are not necessary for flight in the adult but seem to be important for the movements of the nymph^1^,^27^. IIIdlm1 starts out as a broad muscle whose origin covers roughly a fourth of the posterior surface of the intersegmental ridge in the two earlier instars and shrinks to a few fibres in the last instar.

Maloeuf^27^ also stated that nymphs of Odonata have more and larger leg and cervical muscles than the adults 1,20. We can confirm these findings: muscles Idlm1, Idvm10, Itpm10, Itpm11, IIscm7, II (III)spm2, IIvlm7, IIIdlm1, IIIvlm2, IIIvlm4 are present in the nymphs and absent in the adults of *Epiophlebia*^1^.

During ontogenesis the thoracic muscles are, in part, newly formed, transformed or reduced^26^,^27^,^31^. The extent of these modifications seems to be exceptional in Odonata, compared to other non-holometabolous pterygote insects, which usually display a nearly complete set of muscles from the first instar 21,32.

### Muscles missing in *Epiophlebia*

As mentioned above, muscles II (III)dvm1, IIscm2 and IIscm7 are present in Anisoptera but not in *Epiophlebia*. The muscles II (III)dvm1 and IIscm7 are present in Zygoptera and Neoptera^28^ and muscle II (III)dvm1 is also known for Ephemeroptera^32^. This distribution through higher taxa indicates that the presence of these muscles is a plesiomorphic condition for Pterygota. Therefore the missing of these muscles in recent *Epiophlebia* is a derived state. This interpretation may also be true for muscle IIscm2, which, among Odonata, is only present in Anisoptera^20^. Assuming a new formation of this muscle in Anisoptera is not parsimonious, because it is also present in Neoptera^32^,^33^.

Zygoptera have four muscles that are missing in *Epiophlebia* and Anisoptera: II (III)dvm2, Ivlm6 and IIvlm3. These are nymphal muscles in Zygoptera^18^. The muscles Itpm3, Itpm7, Itpm8 and Itpm9 as well as II (III)tpm3 and IIvlm7 are only present in nymphs of Epiprocta. Since Itpm3, II (III)tpm3 and IIvlm7 are missing in Zygoptera^18^ but are present in Neoptera^28^ and IIvlm7 also in Ephemeroptera^32^, it is most parsimonious to assume that their presence is a plesimorphic character for Epiprocta. The muscles II (III)scm1, IIscm8, IIIscm2 and Ispm1 have only been found in nymphs of Odonata. They seem to be generally missing in the adults^1^,^18^,^27^. The odonate ground pattern most likely encompassed IIvlm3 (present in Zygoptera) as well as IIvlm7 (present in Epiprocta), with IIvlm3 secondarily missing in Epiprocta, and IIvlm7 in Zygoptera, because both muscles are present outside of Odonata, i.e. in Neoptera and in Ephemeroptera^28^,^32^.

Asahina^1^ depicted and labelled transverse muscles (II (III)tvm1) in the thorax of adult *E. superstes*. Likewise Maleouf^27^ and Maki^30^ mentioned ventral transverse muscles, and Barlet^34^,^35^ and Matsuda^33^ found them in Zygentoma and in Archaeognatha. None of them named these muscles. Chadwick^36^ gave an overview of the occurrence of ventral transverse muscles in several insect orders. Together with these data, our results clearly indicate that a ventral transverse muscle belongs to the ground pattern of the pterygote thorax. Therefore, the absence of such a muscle in Zygoptera and in Anisoptera suggests that it was lost independently in the last common ancestors of each of these two monophyla, and that only *Epiophlebia* retained this plesiomorphic character.

**Figure 7:**
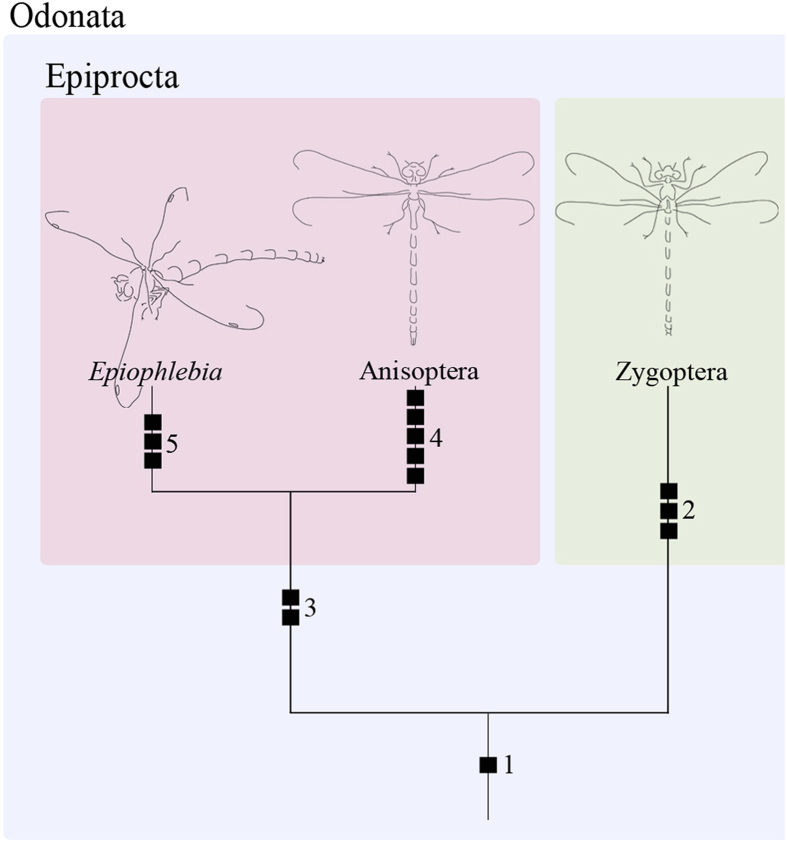
Phylogenetic relationships of Odonata. 1. Ground pattern of Odonata comprising all known odonate muscles. 2. Reduction of II (III)dvm7, II (III)tvm1, II (III)vlm4. 3. Reduction of II (III)tpm2, II (III)dvm2. 4. Reduction of Iscm4, II (III)dvm7, IIscm4, II (III)tvm1, IIIvlm4. 5. Reduction of IIscm2, IIscm7, II (III)dvm1. Drawings by S. Büsse.

Assuming the commonly accepted monophyly of Ptergygota, it is most likely that its last common ancestor was morphologically similar to the extant species of Zygentoma. This also indicates that the number of muscles in the thorax was quite high in the ground pattern, since in Zygentoma and Archaeognatha^33^,^34^,^35^,^36^,^37^ many more thoracic muscles are present than in any extant pterygote insect investigated. This interpretation is supported by our results. We found several muscles in the thorax of Odonata that are not present in Neoptera^28^ (cf. Fig. 7). Among Odonata, species of *Epiophlebia* are those with the highest number of thoracic muscles, indicating that this state is plesiomorphic and that the missing of muscles like Iscm4 & IIIscm4 in Zygoptera or IIscm4 in Anisoptera represent the apomorphic state.

Therefore, the statement of Blanke *et al.*^4^ that *Epiophlebia* has preserved the most ancestral characters in Odoanta is supported.

## Material and Methods

Three different instars (early, middle and last) of both, *E. laidlawi* and *E. superstes*, as well as nymphs of three species of Anisoptera and two species of Zygoptera were investigated (Table 2). The specimens of *E. laidlawi* were fixed in 4% formaldehyde and stored in 80% ethanol. The other specimens were fixed and stored in 80% ethanol. Prior to scanning, the samples were critical point dried (Balzers CPD030) and mounted on facility specific specimen holders. All applicable regulations concerning the protection of free-living species were followed.

As basis for analysing the nymphal morphology and for the three-dimensional reconstructions high resolution X-ray tomography (μCT) datasets^38^ were acquired at the Institut für Paläontologie, University Bonn (Germany) with a GE Phoenix|x-ray v|tome|x tomograph with a 180 kV X-ray source, at the Swiss Light Source, Villigen (Switzerland)^39^ at 10.05 keV and at the Deutsches Elektronen Synchrotron (DESY), Hamburg (Germany)^40^ at 8 keV. Voxel resolutions for the datasets used are given in Table 2. The data were visualized with Amira® 5.4.3 (FEI SAS, Mérignac, France, www.vsg3d.com).

We received the raw tomography data as stacks of TIFF-images containing reconstructed virtual cross-sections (cf. Supplemental Figure 4). Depending on the machine that was used and on the size of the specimen between 800 and 2000 cross-sections were produced per specimen. The TIFF-images were loaded into Amira®, which automatically fuses them into a three-dimensional dataset, which then was stored in the proprietary file-format “.am” that can be processed more easily. To visualize and estimate the quality of the datasets, we first used Amira®’s volume-rendering and section-visualization tools (visualization modules “Volren” and “OrthoSlice”). The set with the best resolution was then chosen to be our point of reference for comparisons with the other specimens. The module “LabelField” was then used to individually label each muscle and the cuticle by scrolling through the slices and marking the relevant structures with either the paint-brush-, lasso- or magic-wand-tool, sometimes in combination with the module’s masking-function. Eventually, we used Amira®’s surface-generation-tools (module “SurfaceGen”) to compute surfaces of the structures of interest: These surfaces can be visualized using the “SurfaceView” module. Images were taken with Amira®’s “SnapShot” function.

These images were further processed (enhancement of brightness/contrast, cropping) with Photoshop® 6.0 (Adobe System Inc., San José, USA). Exemplary sections reconstructed from X-ray tomography data are shown in Supplementary figure 4. A virtual 3D model (produced using Adobe Acrobat Pro® 9.0: “.obj” files of surfaces were exported from Amira®, imported into Adobe 3D Reviewer®, which is part of Acrobat Pro® 9.0, exported to “.u3d” files, which can be inserted into “.pdf”-files) of the thoracic musculature of an *Epiophlebia* nymph is given in Supplementary Figure 5.

## Additional Information

**How to cite this article**: Büsse, S. *et al.* The thorax morphology of *Epiophlebia* (Insecta: Odonata) nymphs – including remarks on ontogenesis and evolution. *Sci. Rep.* **5**, 12835; doi: 10.1038/srep12835 (2015).

## Supplementary Material

Supplementary Information

## Figures and Tables

**Table 1:** Muscles present in the thorax of *Epiophlebia*.

Abbreviation	Name	Origin	Insertion
Prothorax			
Dorsal longitudinal muscles (Fig. 1)			
Idlm1	Musculus prophragma-occipitalis	Apex of tergal apophysis 2	Postoccipital ridge
Idlm3	M. prophragma-cervicalis	Tergal apophysis 1	Tergal apophysis 2
Idlm4	M. cervico-occipitalis dorsalis	Tergal apophysis 1	Post-occipital ridge
Dorsoventral muscles (Fig. 2)			
Idvm10	M. profurca-phragmalis	Apex of profurca	Apex of tergal apophysis 2
Idvm15	M. propleuro-coxalis superior	Anterolateral portion of tergite 1	Anterior base of the procoxa
Idvm18	M. pronto-coxalis lateralis	Postero-lateral portion of tergite 1	Base of the procoxa
Pleuro-coxal muscles (Fig. 3)			
Ipcm8	M. propleuro-trochanteralis	Episternum 1	Tendon of protrochanter
Ipcm9	M. protergro- trochanteralis	Lateral side prothoracic tergite	Tendon of protrochanter
Sterno-coxal muscles (Fig. 4)			
Iscm2	M. profurca-coxalis posterior	Base of profurca	Base of the procoxa
Iscm4	M. profurca-coxalis lateralis	Base of profurca	Procoxa close to interpleural ridge
Iscm6	M. profurca-trochanteralis	Lateral side of profurca	Prothoracic trochanter
Sterno-pleural muscles (Fig. 4)			
Ispm1	M. profurca-apodemalis	Apex of profurca	Prothoracic epimeron
Tergo-pleural muscles (Fig. 5)			
Itpm3	M. pronoto-pleuralis anterior	Lateral side of tergite 1	Episternum 1
Itpm7	M. protergo-cervicalis posterior	Lateral part of tergite 1	Lateral of cervix membrane
Itpm8	M. protergo-cervicalis anterior	Cervical membrane close sternum 1	Postocciput
Itpm9	M. protergo-preepisternalis	Lateral tergite 1	Lateral prothoracic intestine
Itpm10	M. prosterna-coxalis dextra	Ventral prothoracic intestine (dexter)	Procoxal base (sinister)
Itpm11	M. prosterna-coxalis sinister	Ventral prothoracic intestine (sinister)	Procoxal base (dexter)
Ventral longitudinal muscles (Fig. 1)			
Ivlm3	M. profurca-tentorialis	Apex of profurca	Cranial tentorial bar
Ivlm7	M. profurca-mesofurcalis	Profuca	Mesofurca
Mesothorax			
Dorsal longitudinal muscles (Fig. 1)			
IIdlm1	M. prophragma-mesophragmalis	Tergal apophysis 3	Tergal aposphysis 4
Dorsoventral muscles (Fig. 2)			
IIdvm3	M. mesonoto-trochantinalis posterior	Prefurca 2	Antero-lateral edge of mesowing bud
IIdvm4	M. mesonoto-coxalis anterior	Anterio-lateral edge of mesocoxa	Antero-lateral edge of mesowing bud
IIdvm5	M. mesonoto-coxalis posterior	Antero-lateral edge of mesowing bud	Basicoxal ridge 2
IIdvm6	M. mesocoxa-subalaris	Lateral part of tergite 2	Pericoxal membrane
IIdvm7	M. mesonoto-trochanteralis	Antero-median mesothoracic wing bud	Tendon of mesothoracic trochanter
IIdvm8	M. mesofurca-phragmalis	Apex of the mesofurca	Metathoracic tergite
Pleuro-coxal muscles (Fig. 3)			
IIpcm1	M. mesanepisterno-trochantinalis	Pre-episternal sclerite 2	Lateral mesothoracic tergite 2
IIpcm2	M. mesobasalare-trochantinalis	Base of preepisternal apodem 2	Antero-median mesothoracic wing bud
IIpcm4	M. mesanepisterno-coxalis posterior	Katepisternum 2	Antero-external part of mesocoxa
IIpcm6	M. mesopleura-trochanteralis	Dorsal part of Katepisternum 2	Tendon of mesotrochanter
Sterno-coxal muscles (Fig. 4)			
IIscm1	M. mesofurca-coxalis anterior	Lateral base of Mesofurca	Antero-external ridge of mesocoxa
IIscm3	M. mesofurca-coxalis medialis	Base of mesofurca	Margin of mesocoxa
IIscm4	M. mesofurca-coxalis lateralis	Apex of mesofurca	Base of mesocoxa
IIscm6	M. mesofurca-trochanteralis	Latero-external side of mesofurca	Tendon of mesotrochanter
IIscm7	M. mesospina-metacoxalis	Preepisternal apodem 3	Antero-external edge of metacoxa
IIscm8	M. mesospina-mesocoxalis	Preepisternal apodem 3	Pericoxal membrane
Sterno-pleural muscles (Fig. 4)			
IIspm2	M. mesofurca-pleuralis	Apex of mesofurca	Interpleural ridge 2
Tergo-pleural muscles (Fig. 5)			
IItpm3	M. mesonoto-basalaris	Dorsal side of mesowing bud	Ventral side of mesowing bud
IItpm4	M. mesonoto-pleuralis anterior	Dorsal side of mesowing bud	Ventral side of mesowing bud
IItpm6	M. mesonoto-pleuralis posterior	Interpleural ridge 2	Antero-dorsal edge of mesowing bud
IItpm7	M. mesanepisterno-axillaris	Ventral part of epimeron 2	Lateral edge of mesowing bud
IItpm8	M. mesepimero-axillaris secundus	Ventral part of epimeron 2	Lateral edge of mesowing bud
IItpm9	M. mesepimero-axillaris tertius	Dorsal part of epimeron 2	Inner side of ventral portion of mesowing bud
IItpm10	M. mesepimero-subalaris	Interpleural ridge 2	Lateral edge of mesowing bud
Transverso-ventral musculature (Fig. 6)			
IItvm1	M. transverso-mesoventralis	Preepisternal apodeme 2 (dexter)	Preepisternal apodeme 2 (sinister)
Ventral longitudinal muscles (Fig. 1)			
IIvlm1	M. mesospina-metaspinalis	Base of mesofurca	Preepisternal apodem 3
IIvlm6	M. mesospina-abdominosternalis	Preepisternalapodem 3	Antecostal apodem
IIvlm7	M. mesofurca-abdominosternalis	Profurca	Preepisternal apodem 3
Metathorax			
Dorsal longitudinal muscles (Fig. 1)			
IIIdlm1	M. mesophragma-metaphragmalis	Intersgemental ridge	Transversal ridge between abdomen and thorax
IIIdlm2	M. metanoto-phragmalis	Intersgemental ridge	Transversal ridge between abdomen and thorax
Dorsoventral muscles (Fig. 2)			
IIIdvm3	M. metanoto-trochantinalis	Furcasternum 3	Postero-lateral tergite 3
IIIdvm4	M. metanoto-coxalis anterior	Metathoracic wing bud	Basicoxal ridge 3
IIIdvm5	M. metanoto-coxalis posterior	Metacoxaldisk	Antero-lateral edge of metawing bud
IIdvm6	M. metacoxa-subalaris	Apodem tergite 3	Metacoxa
IIIdvm7	M. metanoto-trochanteralis	Metathoracic wing bud	Metathoracic trochanter
IIIdvm8	M. metanoto-phragmalis	Apex of metafurca	Phragma of abdominal segment 2
Pleuro-coxal muscles (Fig. 3)			
IIIpcm1	M. metanepisterno-trochantinalis	Preepisternal sclerite 3	Lateral at tergite 3
IIIpcm2	M. metabasalare-trochantinalis	Preepisternal apodem 3	Metathoracic wing bud
IIIpcm4	M. metanepisterno-coxalis posterior	Katepisternum 3	Antero-external part of metacoxa
IIIpcm6	M. mesopleura-trochanteralis	Katepisternum 3	Tendon of metatrochanter
Sterno-coxal muscles (Fig. 4)			
IIIscm1	M. metafurca-coxalis anterior	Base of Metafurca	Apodeme of metacoxa
IIIscm2	M. metafurca-coxalis posterior	Base of metafurca	Base of metacoxa
IIIscm3	M. metafurca-coxalis medialis	Base of metafurca	Margin of metacoxa
IIIscm4	M. metafurca-coxalis lateralis	Apex of metafurca	Base of metacoxa
IIIscm6	M. metafurca-trochanteralis	Metafurca	Tendon of metatrochanter
Sterno-pleural muscles (Fig. 4)			
IIIspm2	M. metafurca-pleuralis	Apex of metafurca	Interpleural ridge 3
Tergo-pleural muscles (Fig. 5)			
IIItpm3	M. metanoto-basalaris	Dorsal side of metawing bud	Ventral side of metawing bud
IIItpm4	M. metanoto-pleuralis anterior	side of metawing bud	Ventral side of metawing bud
IIItpm6	M. metanoto-pleuralis posterior	Interpleural ridge 3	Metathoracic tergite
IIItpm7	M. metanepisterno-axillaris	Epimeron 3	Metathoracic wing bud
IIItpm8	M. metapimero-axillaris secundus	Epimeron 3	Metathoracic wing bud
IIItpm9	M. metapimero-axillaris tertius	Epimeron 3	Apodeme of tergite 3
IIItpm10	M. metapimero-subalaris	Interpleural ridge 3	Metathoracic wing bud
Transverso-ventral musculature (Fig. 6)			
IIItvm1	M. transverso-mesoventralis	Preepisternal apodeme 3 (dexter)	Preepisternal apodeme 3 (sinister)
Ventral longitudinal muscles (Fig. 1)			
IIIvlm2	M. mesofurca-abdominosternalis	Metafurca	Within the abdomen (second abdominal sternite)
IIIvlm3	M. metaspina-abdominosternalis	Poststernum 3	Within the abdomen (second abdominal sternite)
IIIvlm4	M. abdominosterno-metaspinalis	Cap tendon or apodeme	Within the abdomen (second abdominal sternite)
IIIvlm6	M. mesospina-abdominosternalis	Preepisternal apodem 3	Cap tendon or apodeme

**Table 2:** Specimens investigated and voxel resolution of μCT data. Voxels are isometric in x-, y- and z-axis in all datasets.

Taxa	Species	Instars	Collection	Facility & voxel dimensions	Proposal No.
*Epiophlebia*	*Epiophlebia laidlawi*	2	Hindu Kush Himalayan Benthological Society, Kathmandu, Nepal	SLS 5.92 μm	20080794, Nov. 2010, TH
*Epiophlebia*	*Epiophlebia laidlawi*	1 last instar	Hindu Kush Himalayan Benthological Society, Kathmandu, Nepal	Bonn 25.34 μm	
*Epiophlebia*	*Epiophlebia superstes*	2	Department for Systematic Entomology, Graduate School of Agriculture, Hokkaido University Sapporo, Japan	SLS 5.92 μm	20080794, Nov. 2010, TH
*Epiophlebia*	*Epiophlebia superstes*	1 last instar	Department for Systematic Entomology, Graduate School of Agriculture, Hokkaido University Sapporo, Japan	Bonn 15.1 μm	
Zygoptera	*Ischnura elegans*	1	Zoological Museum, JFB-Institute of Zoology & Anthropology, Georg-August-University Göttingen, Germany	DESY 1.8 μm	I- 20090102, Aug. 2009, SB
Zygoptera	*Nehalennia speciosa*	1	“ ”	SLS 1.85 μm	20080794, May 2009, TH
Anisoptera	*Sympetrum vulgatum*	1	“ ”	DESY 3.6 μm	I- 20090102, Aug. 2009, SB
Anisoptera	*Aeshna affinis*	1	“ ”	SLS 5.92 μm	20100088, Nov. 2010, TH
Anisoptera	*Cordulegaster bidentatus*	1	“ ”	SLS 1.85 μm	20080794, May 2009, TH
