# Supplementary material for: The thorax morphology of Epiophlebia (Insecta: Odonata) nymphs – including remarks on ontogenesis and evolution
Source: Sci Rep. 2015 Aug 6;5:12835. doi: 10.1038/srep12835 (PMC4526886; doi:10.1038/srep12835)
Supplement: Supplementary Information [file srep12835-s1.pdf]

# The thorax morphology of *Epiophlebia* (Insecta: Odonata) nymphs – including remarks on ontogenesis and evolution
# - Supplementary information -

Sebastian Büsse[1], Benjamin Helmker[2] & Thomas Hörnschemeyer[2*]

[1] University Museum of Zoology, Department of Zoology, University of Cambridge, Downing Street, CB2 3EJ Cambridge, UK

[2] J.- F.- Blumenbach Institute for Zoology & Anthropology, Department Morphology, Systematics & Evolutionary Biology Georg-August-University Göttingen, Berliner Str. 28, 37073 Göttingen, Germany

e-mail address: *TH - thoerns@gwdg.de

Büsse, Helmker, Hörnschemeyer: The thorax morphology of *Epiophlebia* (Insecta: Odonata) nymphs – including remarks on ontogenesis and evolution

**Supplementary data 1: List of thoracic muscles of *Epiophlebia* nymphs**

The muscle numbers used by Maloeuf[25] and Asahina[1] are given in paranthesis. An additional number in parentheses within the first set denotes the homologous muscle of the meso- or metathorax. Muscles not recognized by Maloeuf[25] or Asahina[1] are marked with (-). Muscles not mentioned by Friedrich & Beutel[27] are marked with * and named according to their points of origin and insertion.

## Prothorax

**dlm – Dorsal longitudinal muscles** (Figure 1)

**Idlm1**, M. porphragma-occipitalis, (3)

| | |
|---|---|
| Origin: | Apex of the second tergal apophysis |
| Insertion: | Posterior surface of the postoccipital ridge |
| Characteristics: | Idlm1 runs next to the median axis, ventrally to Idlm3. It is responsible for head movements. It is absent in the imago due to Maloeuf[25] but present due to Asahina[1]. |

**Idlm3**, M. prophragma-cervicalis, (2)

| | |
|---|---|
| Origin: | Posterior surface of the first tergal apophysis |
| Insertion: | The second tergal apophysis and intersegmental ridge of the pro- and mesothoracic terga. |
| Characteristics: | Idlm3 proceeds next to the median axis, dorsally to Idlm1. |

**Idlm4**, M. cervico-occipitalis dorsalis, (1)

| | |
|---|---|
| Origin: | Anterior surface of the first tergal apophysis |
| Insertion: | Posterior surface of the post-occipital ridge |
| Characteristics: | Idlm4 runs dorsally along the median axis. It is responsible for head movements. |

**dvm – Dorsoventral muscles** (Figure 2)

**Idvm10**, M. profurca-phragmalis, (20)

| | |
|---|---|
| Origin: | Apex of the profurca |

Insertion: Apex of the second tergal apophysis, which is simultaneously the intersegmental ridge of the pro- and mesothoracic terga[25]

Characteristics: An intersegmental, tergosternal muscle. Idvm10, is absent in the adult1,25.

**Idvm15**, M. pronoto-trochantinocoxalis, (13)

Origin: At a large area of the antero-lateral surface of the prothoracic tergite.

Insertion: The pericoxal membrane at the anterior base of the procoxa.

Characteristics: A large tergocoxal promoter, whose fibre bundles are easily distinguished.

**Idvm18**, M. pronoto-coxalis lateralis, (14) & (15)

Origin: At a large area of the postero-lateral surface of the prothoracic tergite.

Insertion: At the posterior apodeme at the base of the procoxa.

Characteristics: A tergal remoter of the procoxa. Ipcm3 is the largest muscle in the prothorax and its filaments are strikingly separated.

**pcm – Pleuro-coxal muscles** (Figure 3)

**Ipcm8**, M. propleuro-trochantinalis, (18)

Origin: Prothoracic episternum, laterally to Ipcm9.

Insertion: Large tendon attached to the median surface of the prothoracic trochanter.

Characteristics: Pleural depressor of prothoracic trochanter. Ipcm8 is slightly larger than its homologue in the meso- and metathorax.

**Ipcm9**, M. protergo-trochanteralis, (17)

Origin: In the lateral area of the prothoracic tergite, between the filaments of Idvm18.

Insertion: Large tendon attached to the median surface of the prothoracic trochanter.

Characteristics: A large tergo-coxal muscle.

**scm – Sterno-coxal muscles** (Figure 4)

**Iscm2**, M. profurca-coxalis posterior, (16)

Origin: On the lateral surface of the base of the profurca.

Insertion: Postero-median surface of the base of the procoxa.

Characteristics: A flat sterno-coxal muscle.

**Iscm4**, M. profurca-coxalis lateralis, (-)

Origin: Laterally on the apex of the profurca.

Insertion: Laterally on the base of the procoxa, near the interpleural ridge.

Characteristics: A very small sterno-pleural muscle.

**Iscm6**, M. profurca trochanteralis, (19)

Origin: Laterally on the profurca, anterior to Iscm2.

Insertion: Large tendon that is medially attached to the prothoracic trochanter.

Characteristics: A sternal depressor of the prothoracic trochanter.

**spm – Sterno-pleural muscles** (Figure 4

**Ispm1**, M. profurca-occpitalis, (-)

Origin: Lateral surface of the apex of the profurca.

Insertion: Antero-lateral area of the prothoracic epimeron.

Characteristics: A short sterno-pleural muscle, which resembles IIspm2 and IIIspm2 in shape and point of origin. It runs diagonally between Idvm15 and Ipcm9.

**tpm – Tergo-pleural muscles** (Figure 5)

**Itpm3**, M. pronoto-pleuralis anterior, (12)

Origin: Lateral area of the prothoracic tergite, just postero-lateral to Itpm9.

Insertion: Anterior part of the prothoracic episternum, just posterior of the cervical membrane and postero-dorsal of Itpm7.

Characteristics: A small, tergo-pleural muscle1,25.

**Itpm7**, M. protergo-cervicalis posterior, (4)

Origin: Lateral area of the prothoracic tergite.

Insertion: Lateral cervixcal membrane.

Characteristics: Itpm7 runs tergo-pleural in the nymph and arches anteriorly around Itpm9.

**Itpm8**, M. protergo-cervicalis anterior, (5)

Origin: Cervical membrane on the antero-lateral of the prothoracic sternum.

Insertion: Postocciput, anterior to Itpm7.

Characteristics: Itpm8 runs tergo-sternal and is the most anterior muscle of the prothorax.

**Itpm9**, M. protergo-preepisternalis, (7)

Origin: Lateral area of the prothoracic tergite, postero-lateral to Itpm7.

Insertion: Attaches laterally on the prothoracic intestine, at height of the dorso-ventral axis.

Characteristics: Itpm9 runs medially of Itpm7 and Itpm8.

**Itpm10**, M. prosterna-coxalis dextra, (9)

Origin: Ventral surface of the prothoracic intestine.

Insertion: Anterior margin of left coxal base.

Characteristics: Itpm10 is a very thin muscle that can only be traced in the youngest nymph. It is absent in the imago due to Maloeuf[25] but present due to Asahina[1] .

**Itpm11**, M. prosterna-coxalis sinister, (10)

Origin: Ventral surface of the prothoracic intestine.

Insertion: Anterior margin of right coxal base.

Characteristics: Itpm11 is a very thin muscle that can only be traced in the youngest nymph. It is absent in the imago due to Maloeuf[25] but present due to Asahina[1].

**vlm – Ventral longitudinal muscles** (Figure 1)

**Ivlm3**, M. profurca-tentorialis, (11) & (11')

Origin: Apex of the profurca.

Insertion: Cranial tentorial bar.

Characteristics: A sternal longitudinal muscle, responsible for head movements. According to Malouef[25], there is another muscle, (11'), running ventrally of Ivlm3, originating from the apex of the profurca as well, but inserting on the membrane ventral to the tentorial bar. The muscle (11') was not found in any of the specimens.

**Ivlm7**, M. profurca-mesofurcalis, (41)

Origin: Posterior surface of the profuca.

Insertion: Anterior surface of the mesofurca.

Characteristics: Ivlm7 splits into two filaments, connecting either bases or apexes of the meso- and profurca. It is listed as muscle (42) by Maloeuf[25].

## Mesothorax

**dlm – Dorsal longitudinal muscles** (Figure 1)

**IIdlm1**, M. prophragma-mesophragmalis, (25 (45))

Origin: Postero-lateral surface of the third tergal apophysis.

Insertion: Antero-lateral surface of the fourth tergal apophysis.

Characteristics: A small, flat muscle.

**dvm – Dorsoventral muscles** (Figure 2)

**IIdvm3**, M. mesonoto-trochantinalis posterior, (23 (46))

Origin: Mesothoracic furcasternum.

Insertion: On the antero-ventral edge of the mesothoracic wing bud.

Characteristics: In the adult it originates from the mesothoracic prefurca and is an elevator of the adult's forewing. The prefurcal ridges are not developed in the examined instars. Its diameter increases during the nymphal development.

**IIdvm4**, M. mesonoto-coxalis anterior, (26 (48))

Origin: Antero-median surface inside the mesothoracic wing bud, opposite of IIdvm3.

Insertion: Lateral surface of the mesothoracic basicoxal ridge.

Characteristics: A future elevator of the forewing. IIdvm4 is the thinnest muscle in the mesothorax. It arches anteriorly around IIspm2. IIdvm4 does not split into two strands in *E. laidlawi* and *E. superstes* as stated by Maloeuf[25] for the dragonflies.

**IIdvm5**, M. mesonoto-coxalis posterior, (27 (49))

Origin: On the anterior ventral edge of the mesothoracic wing bud, just ventro-laterally of IIdvm4.

Insertion: Lateral surface of the basicoxal ridge, posteriorly to IIdvm4.

Characteristics: An elevator of the adult's forewing. IIdvm5 arches posteriorly around IIspm2 and is attached very closely to IIdvm4.

**IIdvm6**, M.mesocoxa subalaris, (37 (60))

Origin: Latero-sternal area of the mesothoracic tergite, just posteriorly to IIIdvm3, ventrally to IIIdvm4 and IIIdvm5.

Insertion: Apodeme attached to the postero-lateral side of the pericoxal membrane.

Characteristics: IIdvm6 is the largest muscle in the mesothorax. It is a tergal remoter of the mesocoxa.

**IIdvm7**, M. mesonoto-trochanteralis, (-)

Origin: Antero-median surface inside the mesothoracic wing bud, just anteriorly to IIdvm4.

Insertion: The large tendon attached to the median side of the mesothoracic trochanter.

Characteristics: A long tergo-coxal muscle, which runs parallel to IIdvm4 at first, then comes apart at around half its course and pulls through the coxa.

**IIdvm8**, M. mesofurca-phragmalis, ( - (20, 67))

Origin: Apex of the mesofurca.

Insertion: Laterally on the most antero-lateral portion of the metathoracic tergite, just anteriorly to IIIpcm1.

Characteristics: A long and thin dorso-sternal intersegmental muscle. Its homologues are IIdvm10 in the prothorax and IIIdvm8 in the metathorax.

**pcm – Pleuro-coxal muscles** (Figure 3)

**IIpcm1**, M. mesanepisterno-trochantinalis, (21 (43))

Origin: Mesothoracic preepisternal sclerite, anteriorly of the mesocoxa.

Insertion: Antero-lateral side of the mesothoracic tergite.

Characteristics: A depressor of the imago's forewing. The cap tendon described by Asahina[1] is not visible. IIpcm1 is listed as Muscle (22) by Maloeuf[25].

**IIpcm2**, M. mesobasalare-trochantialis, (22 (44))

Origin: At the base of the mesothoracic preepisternal apodem, close to the lateral ridge.

Insertion: Antero-median area of the inner, dorsal surface of the mesothoracic wing bud, dorsally of IIpcm1.

Characteristics: A future depressor of the forewing. The cap tendon described by Asahina[1] is not visible. IIpcm2 is the only muscle originating from this region and therefore distinctive. This muscle starts out small in the earliest instar, but grows to one of the largest muscles in the latest instar. IIpcm2 is listed as

Muscle (21) by Maloeuf[25].

**IIpcm4**, M. mesanepisterno-coxalis posterior, (36 (58))

Origin: Dorsal portion of the mesothoracic katepisternum.

Insertion: Apodeme on the antero-external side of the mesocoxa.

Characteristics: A large fan-shaped muscle and the pleural promoter of the mesocoxa.

**IIpcm6**, M. mesopleuro-trochanteralis, (39 (62))

Origin: Dorsal portion of the mesothoracic katepisternum.

Insertion: Large tendon attached to the base of the mesothoracic trochanter, laterally to Idvm7 and IIscm6.

Characteristics: Pleural depressor of the trochanter. IIpcm6 and IIpcm4 originate very close to each other, with IIpcm6 on the median side.

**scm – Sterno-coxal muscles** (Figure 4)

**IIscm1**, mesofurca-coxalis anterior, (-)

Origin: Anterio-laterally to the base of the mesofurca.

Insertion: Apodeme on the antero-external side of the mesocoxa, just postero-laterally to IIpcm4.

Characteristics: A very small muscle running anteriorly and parallel to IIscm6.

**IIscm3**, M. mesofurca-coxalis medialis, (38 (61))

Origin: Inner surface of the base of the mesofurca.

Insertion: Postero-internal margin of the mesocoxa, just medially of IIdvm6.

Characteristics: The sternal remoter of the mesocoxa. IIscm3 does insert very closely to the same apodeme as IIdvm6.

**IIscm4**, M. mesofurca-coxalis lateralis, (-)

Origin: Lateral surface of the apex of the mesofurca.

Insertion: Laterally on the base of the mesocoxa, near the interpleural ridge.

Characteristics: A very small muscle.

**IIscm6**, M. mesofurca-trochanteralis, (40 (63))

Origin: Latero-external surface of the mesofurca.

Insertion: Large tendon attached to the base of the mesothoracic trochanter, medially to IIdvm7 and IIscm6.

Characteristics: The largest sterno-trochanternal muscle in the nymph, covering almost the whole lateral surface of the mesofurca.

**IIscm7**, M. mesospina-metacoxalis, (59)

Origin: Metathoracic preepisternal apodeme.

Insertion: Antero-external rim of metacoxa.

Characteristics: A sternal promoter of the metacoxa. IIscm7 has no homologue in the mesothorax and is absent in the adult[1,22,25].

**IIscm8**, M. mesospina-mesocoxalis, (-)

Origin: Medio-ventral surface of the metathoracic preepisternal apodeme.

Insertion: Apodeme attached to the postero-lateral pericoxal membrane, next to IIdvm6.

Characteristics: A fusiform, intersegmental muscle with no homologue in the metathorax.

**spm – Sterno-pleural muscles** (Figure 4)

**IIspm2**, M. mesofurca-pleuralis, (35 (57))

Origin: Apex of the mesofurca.

Insertion: Apex of mesothoracic interpleural ridge.

Characteristics: A flat, fan-shaped muscle that runs diagonally between IIdvm4 and IIdvm5. It is absent in the adult[1,22,25].

**tpm – Tergo-pleural muscles** (Figure 5)

**IItpm3,** M. mesonoto-basalaris, (-)

Origin: Antero-laterally on the dorsal surface inside the mesothoracic wing bud.

Insertion: Antero-laterally on the ventral surface inside the metathoracic wing bud.

Characteristics: IItpm3 is a small muscle that will be turned upside-down in the imago. It lies antero-laterally of IItpm4.

**IItpm4**, M. mesonoto-pleuralis anterior, (28 (50))

Origin: Dorsal surface inside the mesothoracic wing bud.

Insertion: Ventral surface inside the mesothoracic wing bud.

Characteristics: IItpm4 is a small muscle that will be turned upside-down in the imago.

**IItpm6**, M. mesonoto-pleuralis posterior, (31 (53))

Origin: Upper portion of mesothoracic interpleural ridge.

Insertion: On the antero-ventral edge of the mesothoracic wing bud, just anteriorly to IIdvm1.

Characteristics: A short tergo-pleural muscle.

**IItpm7**, M. mesanepisterno-axillaris, (33 (55))

Origin: Ventral portion of mesothoracic epimeron.

Insertion: Lateral portion of the antero-dorsal edge of the mesothoracic wing bud, just posteriorly to IItpm8.

Characteristics: A future depressor of the forewing. IItpm7 does not originate from a small cap-tendon[1]. It is parallel to IItpm8. Especially in the oldest nymph both increase their size and seem to merge.

**IItpm8**, M. mesepimero-axillaris secundus, (32 (54))

Origin: Lateral area of the antero-dorsal edge of the mesothoracic wing bud.

Insertion: Ventral area of mesothoracic epimeron.

Characteristics: A future depressor of the forewing.

**IItpm9**, M. mesepirmero-axillaris tertius, (29 (51)) & (30 (52))

Origin: Dorso-lateral portion of the mesothoracic epimeron, just posteriorly to IItpm6's origin.

Insertion: Inner surface of the most ventro-lateral extension of the mesothoracic wing bud.

Characteristics: A short and thick muscle, becoming a depressor of the forewing. A membrane as described by Asahina[1] is not visible. Also, a further muscle (30) that is supposed to run ventrally of IItpm9 is not visible.

**IItpm10**, M. mesepimero-subalaris, (34 (56))

Origin: On the mesothoracic interpleural ridge.

Insertion: Lateral area of the antero-dorsal margin of the mesothoracic wing bud, just posterior to IItpm7.

Characteristics: A future depressor of the forewing. IItpm10 does not originate from a cap-tendon[1], but very close to IItpm7 and IItpm8.

**tvm – Transverso-ventral musculature** (Figure 6)

**IItvm1***, M. transverso-mesoventralis, (-)

Origin: Medially on the right metathoracic preepisternal apodeme.

Insertion: Medially on the left metathoracic preepisternal apodeme.

Characteristics: The only transverse muscle in the metathorax. IIItvm1 runs directly above the nervous system parallel to the ventral intersegmental ridge. Depicted but not described by Maloeuf[25] and Asahina[1]; not mentioned by other authors[22,26].

**vlm – Ventral longitudinal muscles** (Figure 1)

**IIvlm1,** M. mesospina-metaspinalis

Origin: Base of mesofurca.

Insertion: Base of preepisternal apodem.

Characteristics: This muscle runs between IIvlm7 and IIvlm6.

**IIvlm6**, M. mesospina-abdominosternalis (68)

Origin: Posterior surface of preepisternal apodem.

Insertion: Antecostal apodem of the first abdominal segment.

Characteristics: This muscle runs from the mesothorax into the abdomen.

**IIvlm7**, M. mesofurca-abdominosternalis, (42)

Origin: Posterior surface of profurca.

Insertion: Anterior surface of metathoracic preepisternal apodeme.

Characteristics: A broad muscle that runs around, but is not attached to the median surface of the mesofurca. IIvlm7 (42) is absent in the imago1,22 and listed as muscle (41) by Maloeuf[25].

## Methathorax

**dlm – Dorsal longitudinal muscles** (Figure 1)

**IIIdlm1**, M. mesophragma-metaphragmalis, (45 (25))

Origin: Postero-lateral surface of the intersegmental ridge between the meso- and

metathoracic terga.

Insertion: Lateral portion of the thoracic-abdominal transverse ridge.

Characteristics: IIIdlm1 decreases its size during the nymphal development. It starts out as a broad muscle covering roughly a fourth of the intersegmental ridge's posterior surface in the two earlier nymphs and shrinks to a small string in the latest. IIIdlm1 is absent in the adult[1,22,25].

**IIIdlm2**, M. metanoto-phragmalis, (45')

Origin: Most lateral area of the posterior surface of the intersegmental ridge between the meso- and metathoracic terga.

Insertion: Lateral area of the thoracic-abdominal transverse ridge.

Characteristics: Runs laterally and parallel to IIIdlm1.

**dvm – Dorsoventral muscles** (Figure 2)

**IIIdvm3**, M. metanoto-trochantinalis, (46 (23))

Origin: Metathoracic furcasternum.

Insertion: Postero-laterally on the most antero-lateral area of the metathoracic tergite, just postero-dorsally to IIIpcm1.

Characteristics: An elevator of the imago's hind wing. In the adult the origin of this muscle will be on the then larger metathoracic prefurcal ridge. It gains diameter in the later nymph but does not exceed the size of its homologue in the mesothorax.

**IIIdvm4**, M. metanoto-pleuralis anterior, (48 (26))

Origin: Antero-internal surface of the metathoracic wing bud.

Insertion: Lateral surface of the basicoxal ridge.

Characteristics: An elevator of the adult's hind wing. IIdvm4 is the thinnest muscle in the metathorax. It runs parallel to IIIdvm7 and arches around IIIspm2 anteriorly.

**IIIdvm5**, M. metanoto-coxalis posterior, (49 (27))

Origin: Anterior margin of the lateral apodeme of the metathoracic tergite, just posteriorly of IIIdvm4.

Insertion: Lateral area of the basicoxal ridge, next to IIIdvm4.

Characteristics: An elevator of the future hind wing. IIIdvm5 arches posteriorly around

IIIdvm6 and is attached next to IIIdvm4.

**IIIdvm6**, M. metacoxa-subalaris, (60 (37))

Origin: Ventral surface of the most lateral apodeme of the metathoracic tergite.

Insertion: Disc interior to coxal condyle in the lateral area of the metacoxa.

Characteristics: The largest muscle in the metathorax and a tergal remoter of the metacoxa.

**IIIdvm7**, M. metanoto-trochanteralis, (-)

Origin: Antero-ventral edge of the metathoracic wing bud, just antero-ventrally to IIIdvm4.

Insertion: Through a tendon attached to the median margin of the metathoracic trochanter.

Characteristics: A long tergo-coxal muscle, that runs parallel to IIIdvm4 at first, then comes apart at around half its course and pulls through the coxa.

**IIIdvm8**, M. metafurca-phragmalis, (67, (-, 20))

Origin: Apex of the metafurca.

Insertion: Ventro-lateral area of the phragma anterior to the second abdominal segment.

Characteristics: IIIdvm8 is a flat, fan-shaped intersegmental muscle. Its homologue in the mesothorax is IIdvm8, in the prothorax it is Idvm10. The latter two are of different shapes, but originate and insert on similar structures.

**pcm – Pleuro-coxal muscles** (Figure 3)

**IIIpcm1**, M. metanepisterno-trochantinalis, (43 (21))

Origin: The metathoracic preepisternal sclerite.

Insertion: Laterally on the most antero-lateral area of the metathoracic tergite.

Characteristics: A future elevator of the hind wing. IIIpcm1 originates from a very lateral area of the metathoracic preepisternal sclerite, anteriorly of the coxal condyle. It is listed as Muscle 44 by Maloeuf[25].

**IIIpcm2**, M. metabasalare-trochantinalis, (44 (22))

Origin: Metathoracic preepisternal apodeme.

Insertion: Antero-median area of the inner dorsal surface of the metathoracic wing

bud, dorsally of IIIpcm1.

Characteristics: A depressor of the imago's hind wing. IIIpcm2 and IIIpcm1 do not have a joint point of insertion as described by Asahina[1]. IIIpcm2 is the only tergo-pleural muscle to originate from the metathoracic preepisternal apodeme and therefore distinctive. IIIpcm2 becomes larger and flatter in the later instars, covering more area at its point of origin. It is the largest muscle in the later nymph's metathorax, outgrowing even IIIdvm6. IIIpcm2 is listed as Muscle (43) by Maloeuf[25].

**III pcm4**, M. metaepisterno-coxalis posterior, (58 (36))

Origin: Dorsal area of the metathoracic katepisternum.

Insertion: On the antero-external apodem of the metacoxa.

Characteristics: A large fan-shaped muscle and the pleural promoter of the metacoxa.

**IIIpcm6**, M. metapleura-trochanteralis, (62 (39))

Origin: Dorsal area of the metathoracic katepisternum.

Insertion: Attached to the base of the metathoracic trochanter through a tendon.

Characteristics: Pleural depressor of the metathoracic trochanter. IIIpcm6 and IIIpcm4 originate next to each other, with IIpcm6 on the median side.

Characteristics: A very small muscle that runs anteriorly of and parallelly to IIIscm6.

**scm – Sterno-coxal muscles** (Figure 4)

**IIIscm1**, M. metafurca-coxalis anterior, (-)

Origin: Anterio-laterally to the base of the metafurca.

Insertion: On an apodeme at the antero-lateral inner surface of the metacoxa, just postero-laterally to IIIpcm4.

Characteristics: A very small muscle, which runs anteriorly and parallelly to IIIscm6.

**IIIscm2**, M. metafurca-coxalis posterior, (-)

Origin: Posterior surface at the base of the metafurca.

Insertion: Posterio-medially at the base of the metacoxa, next to IIIdvm6.

Characteristics: A large, flat sterno-coxal muscle.

**IIIscm3**, M. metafurcacoxalis medialis, (61 (38))

Origin: Inner surface of the base of the metafurca.

Insertion: Postero-internal margin of the metacoxa, just medially of IIIdvm6.

Characteristics: The sternal remoter of metacoxa.

**IIIscm4**, M. metafurca-coxalis lateralis, (-)

Origin: Lateral surface of the apex of the metafurca.

Insertion: Lateral surface of the base of the metacoxa, close to the interpleural rige.

Characteristics: A very small muscle.

**IIIscm6**, M. metafurca-trochanteralis, (63 (40))

Origin: Antero-lateral area of the metafurca, anterior to IIIscm3.

Insertion: Attached to the base of the metathoracic trochanter through a tendon.

Characteristics: The sternal depressor of the trochanter.

**spm – Sterno-pleural muscles** (Figure 4)

**IIIspm2**, M. mesofurca-pleuralis, (57 (35))

Origin: Apex of metafurca.

Insertion: Interpleural ridge.

Characteristics: A flat, fan-shaped muscle that runs diagonally between IIIdvm4 and IIIdvm5. It is absent in the adult[1,22,25].

**tpm – Tergo-pleural muscles** (Figure 5)

**IIItpm3,** M. metanoto-basalaris, (-)

Origin: Antero-laterally on the antero-dorsal margin of the metathoracic wing bud.

Insertion: Antero-medially on the antero-ventral margin of the metathoracic wing bud.

Characteristics: IIItpm3 is a small muscle that will be turned upside-down in the imago. It runs antero-medially of IIItpm4.

**IIItpm4**, M. metanoto-pleuralis anterior, (50 (28))

Origin: Dorsal surface inside the metathoracic wing bud.

Insertion: Ventral surface inside the metathoracic wing bud.

Characteristics: IItpm4 is a small muscle that will be turned upside-down in the imago.

**IIItpm6**, M. metanoto-pleuralis posterior, (53 (31))

Origin: Upper area of the metathoracic interpleural ridge.

Insertion: Laterally on the most antero-lateral area of the metathoracic tergite, just dorsally to IIIpcm1.

Characteristics: A short, tergo-pleural muscle. IIItpm6 is absent in the adult[1,22,25].

**IIItpm7**, M. metanepisterno-axillaris, (55 (33))

Origin: Ventral area of metathoracic epimeron.

Insertion: Lateral area of the antero-dorsal margin of the metathoracic wing bud.

Characteristics: A depressor of the imago's hind wing. IIItpm7 originates directly from the membrane posterior to the axillary plate and not from a cap-tendon attached to the membrane as described by Asahina[1]. It is parallel to IItpm8. Both increase their size during nymphal development and seem to merge.

**IIItpm8**, M. metepimero-axillaris secundus, (54 (32))

Origin: Ventral area of metathoracic epimeron.

Insertion: Lateral area of the antero-dorsal margin of the metathoracic wing bud.

Characteristics: A future depressor of the hind wing.

**IIItpm9**, M. metapimero-axillaris tertius, (51 (29)) & (52 (30))

Origin: Dorso-lateral area of the metathoracic epimeron, just posterior to IIItpm6.

Insertion: Dorsal surface of the lateral apodeme of the metathoracic tergite.

Characteristics: A depressor of the adult's hind wing. The muscle (52) as described by Asahina[1] is not visible.

**IIItpm10**, M. metepimero-subalaris, (56 (34))

Origin: On the ventral half of the interpleural ridge.

Insertion: Lateral area of the antero-dorsal margin of the metathoracic wing bud.

Characteristics: Future depressor of the hind wing. IIItpm10 does not originate from a cap-tendon attached to the membrane posterior to the axillary plate as described by Asahina[1]. It shares its point of origin with IIItpm7 and IIItpm8.

**tvm – Transverso-ventral musculature** (Figure 6)

**IIItvm1***, M. transverso-metaventralis, (-)

Origin: Medially on the right metathoracic preepisternal apodeme.

Insertion: Medially on the left metathoracic preepisternal apodeme.

Characteristics: The only transverse muscle in the metathorax. IIIvtm1 runs directly above the nervous system, parallelly to the ventral intersegmental ridge. Not mentioned in[1,22,25,26].

**vlm – Ventral longitudinal muscles** (Figure 1)

**IIIvlm2**, M. metafurca-abdominosternalis, (65)

Origin: Posterior surface of the metafurca.

Insertion: Ventral area of the membrane anteriorly to the second abdominal sternite.

Characteristics: A small muscle that winds around a tendon, attached to IIIvlm4. It is absent in the imago[1,25].

**IIIvlm3**, M. metaspina-abdominosternalis, (66)

Origin: In a large area of the metathoracic poststernum.

Insertion: Antero-ventral membrane of the second abdominal sternite.

Characteristics: A bulky, fibred muscle.

**IIIvlm4***, M. abdominosterno-metaspinalis, (64)

Origin: Via a cap tendon attached to the anterior phragma of the first abdominal segment.

Insertion: Antero-ventral process of second abdominal sternite, dorsally to IIIvlm3.

Characteristics: IIIvlm4 is the last muscle in a line consisting of IIvlm7 (42), IIIvlm6 (68) and IIIvlm4 (64). These three muscles connect the mesofurca, the metathoracic preepisternal apodeme and the abdomen. It is absent in the imago[1,22,25].

**IIIvlm6**, M. mesospina-abdominosternalis, (68)

Origin: Metathoracic preepisternal apodeme.

Insertion: Attached to the anterior phragma of the first abdominal segment through a cap-tendon.

Characteristics: IIvlm6 is a fan-shaped longitudinal muscle. At its point of origin, it is as broad and flat as IIvlm7 in the mesothorax. It becomes a narrow cone at its insertion.

**Supplementary table 1**: Homologisation of thoracic muscle nomenclatures used by several authors
"-" absent / "?" uncertain or no information

| Friedrich & Beutel (2008) | this study | Büsse & Hörnschemeyer (2013) | Büsse et al. (2013) | Asahina (1954) | Willkommen (2008) | Wittig (1955) | Matsuda (1970) |
|---|---|---|---|---|---|---|---|
| **Prothorax** | | | | | | | |
| Idlm1 | x | x | ? | 3 | ? | I dlm 10 | op-t 3 |
| Idlm2 | - | - | ? | - | ? | 0 dlm 1 | op-t 2 |
| Idlm3 | x | x | ? | 2 | ? | I dlm 11b | cv(d)-t 1, t 14 |
| Idlm4 | x | x | ? | 1 | ? | 0 dlm 2 | op-t 1 |
| Idlm5 | - | - | ? | - | ? | I dlm 12 | t 12 |
| Idlm6 | - | - | ? | - | ? | I dlm 12? | t 13 |
| Idvm1 | - | - | ? | - | ? | 0 lm 7 | op-cv 1 |
| Idvm2 | - | - | ? | - | ? | 0 lm 7 | op-cv 2 |
| Idvm3 | - | - | ? | - | ? | 0 lm 8 | op-cv 3 |
| Idvm4 | - | - | ? | - | ? | 0 lm 5 | t-s(cv) 1? |
| Idvm5 | - | - | ? | - | ? | - | t-cv 1 |
| Idvm6 | - | - | ? | - | ? | 0 lm 6 | t-cv 2 |
| Idvm7 | - | - | ? | - | ? | - | t-cv 3 |
| Idvm8 | - | - | ? | - | ? | - | t-s(cv) 9 |
| Idvm9 | - | - | ? | - | ? | - | op-s 2, p-s 3 |
| Idvm10 | x | x | ? | - | ? | I ism 22 | t-s 1 |
| Idvm11 | - | - | ? | - | ? | I ism 24 | t-s 8 |
| Idvm12 | - | - | ? | - | ? | - | t-s 2 |
| Idvm13 | - | - | ? | - | ? | I dvm 15 | t-ti(cx) 2 |
| Idvm14 | - | - | ? | - | ? | I dvm 16 | t-ti(cx) 3 |
| Idvm15 | x | x | ? | 13 | ? | I dvm 17? | t-ti(cx) 1 |
| Idvm16 | - | - | ? | - | ? | I dvm 19 | t-cx 5 |
| Idvm17 | - | - | ? | - | ? | I dvm 20 | t-cx 6, t-cx 7 |
| Idvm18 | x | x | ? | 14 & 15 | ? | I dvm 21 | t-cx 8 |
| Idvm19 | - | - | ? | - | ? | I dvm 18 | t-tr 1 |
| Itpm1 | - | - | ? | - | ? | - | op-p 2 |
| Itpm2 | - | - | ? | - | ? | 0 lm 9 | op-p 1, t-p 3 |
| Itpm3 | x | x | ? | 12 | ? | I tpm 25 | - |
| Itpm4 | - | - | ? | - | ? | I tpm 26 | t-p 14? |
| Itpm5 | - | - | ? | - | ? | I tpm 27? | t-p 15? |
| Itpm6 | - | - | - | ? | - | ? | - | t-p 1, t-p 2 |
| - | x | Itpm7 | ? | 4 | ? | ? | ? |
| - | x | Itpm8 | ? | 5 | ? | ? | ? |

Supplementary table 1: Homologisation of thoracic muscle nomenclatures used by several authors
"-" absent / "?" uncertain or no information

| | | | | | | | |
|---|---|---|---|---|---|---|---|
| - | x | Itpm9 | ? | 7 | ? | ? | ? |
| - | x | Itpm10 | ? | 9 | ? | ? | ? |
| - | x | Itpm11 | ? | 10 | ? | ? | ? |
| Ispm1 | x | x | ? | - | ? | I zm 34 | p-s1 |
| Ispm2 | - | - | ? | - | ? | - | p-s 2 |
| Ispm3 | - | - | ? | - | ? | - | p-s 6 |
| Ispm4 | - | - | ? | - | ? | - | p-s 7 |
| Ispm5 | - | - | ? | - | ? | - | p-s 5 |
| Ispm6 | - | - | ? | - | ? | - | p-s 4 |
| Ispm7 | - | - | ? | - | ? | - | p-s 10 |
| Ipcm1 | - | - | ? | - | ? | - | cv-cx 3 |
| Ipcm2 | - | - | ? | - | ? | - | cv-cx 1, cv-cx 2 |
| Ipcm3 | - | - | ? | - | ? | - | p-ti(cx) 1 |
| Ipcm4 | - | - | ? | - | ? | - | p-cx 4 |
| Ipcm5 | - | - | ? | - | ? | I cpm 28 | p-cx 5 |
| Ipcm6 | - | - | ? | - | ? | - | p-cx 6, p-cx 9 |
| Ipcm7 | - | - | ? | - | ? | - | p-cx 7 |
| Ipcm8 | x | x | ? | 18 | ? | I cpm 29 | p-tr 1, p-tr 2 |
| - | x | Ipcm9 | ? | 17 | ? | ? | ? |
| Ivlm1 | - | - | ? | - | ? | 0 vlm 4 | cv-s 1, cv-s 4? |
| Ivlm2 | - | - | ? | - | ? | - | op-cv(v) 4 |
| Ivlm3 | x | x | ? | 11 | ? | 0 vlm 3 | s 1, s 2 |
| Ivlm4 | - | - | ? | - | ? | I vlm 14 | s 14, s16 |
| Ivlm5 | - | - | ? | - | ? | - | s 17 |
| Ivlm6 | - | - | ? | - | ? | - | s 15 |
| Ivlm7 | x | x | ? | 41 | ? | I vlm 13 | s 13 |
| Ivlm8 | - | - | ? | - | ? | - | s 11 |
| Ivlm9 | - | - | ? | - | ? | - | s 12 |
| Iscm1 | - | - | ? | - | ? | I bm 30 | s-cx 5 |
| Iscm2 | x | x | ? | 16 | ? | I bm 33 | s-cx 3 |
| Iscm3 | - | - | ? | - | ? | I bm 32 | s-cx 6 |
| Iscm4 | x | - | ? | - | ? | - | s-cx 2 |
| Iscm5 | - | - | ? | - | ? | - | s-cx 4 |
| Iscm6 | x | x | ? | 19 | ? | I bm 31 | s-tr 1 |
| Iscm7 | - | - | ? | - | ? | - | s-cx 1, s-cx 7 |

Supplementary table 1: Homologisation of thoracic muscle nomenclatures used by several authors
"-" absent / "?" uncertain or no information

| Mesothorax | | | | | | | |
|---|---|---|---|---|---|---|---|
| IIdlm1 | x | x | x | 25 | MT.m | II dlm 35 | t 14 |
| IIdlm2 | - | - | - | - | S.LP?m | II dlm 36, II dlm 37 | t12, t13 |
| IIdlm3 | - | - | - | - | S.Esm | - | t-p 5, t-p 6 |
| IIdvm1 | - | x | x | 23' | S.CmA | II dvm 40 | t-ti 1, t-ti 2 |
| IIdvm2 | - | - | - | - | S.CmA | II dvm 41 | t-ti 3 |
| IIdvm3 | x | x | x | 23 | - | - | t-cx 5 |
| IIdvm4 | x | x | x | 26 | PSL.Cm, S.CmP | II dvm 43 | t-cx 6, t-cx 7 |
| IIdvm5 | x | x | x | 27 | SA.Cm, SA.Fm | II dvm 43 | t-cx 8 |
| IIdvm6 | x | x | - | - | S.Trm | II cpm 53 | t-tr 1 |
| IIdvm7 | x | - | - | - | - | II dvm 42 | t-s 1 |
| IIdvm8 | - | - | - | - | - | II ism 44 | t-s 8, t-s 7 ? |
| IIdvm9 | - | - | - | - | A?.Pm | - | t-p 3 |
| IItpm1 | - | - | - | - | BA.Pm | II tpm 46a | t-p 4, t-p 20 |
| IItpm2 | - | - | x | - | - | II tpm 47 | t-p 7, t-p 8, t-p 9 |
| IItpm3 | x | x | - | - | SrA.Pm, Ax.Pml | II tpm 46b ? | t-p 10, t-p 11, t-p 18 |
| IItpm4 | x | x | x | 28 | - | - | t-p 12 |
| IItpm5 | - | - | - | - | - | - | t-p 15 |
| IItpm6 | x | x | x | 31 | - | II tpm 49 | t-p 13 |
| IItpm7 | x | x | x | 33 | - | II tpm 48 | - |
| IItpm8 | x | x | x | 32 | Ax.PmS | - | t-p 14 |
| IItpm9 | x | x | x | 29/30 | - | - | t-p 16 |
| IItpm10 | x | x | x | 34 | - | II ppm 56 | t-p 19 |
| IItpm11 | - | - | - | - | - | - | t-p 17 |
| IItpm12 | - | - | - | - | - | - | p 1 |
| IItpm13 | - | - | - | - | - | ? | ? |
| IItvm1 | x | - | - | x (?) | - | - | - |
| IIppm1 | - | - | - | - | - | II im 65a | p 2 |
| IIppm2 | - | - | - | - | - | II pm 54a, b | p 3 |
| IIspm1 | - | - | - | - | - | II ppm 55 | p-s 1 |
| IIspm2 | x | x | - | 35 | - | II zm 61a | p-s 2 |
| IIspm3 | - | - | - | - | - | - | p-s 6 |
| IIspm4 | - | - | - | - | - | - | p-s 7 |
| IIspm5 | - | - | - | - | - | - | p-s 3 |
| IIspm6 | - | - | - | - | - | - | p-s 9 |

Supplementary table 1: Homologisation of thoracic muscle nomenclatures used by several authors
"-" absent / "?" uncertain or no information

| | | | | | | | |
|---|---|---|---|---|---|---|---|
| IIspm7 | - | - | - | - | - | - | p-s 5 |
| IIspm8 | - | - | - | - | - | - | p-ti(cx) 1 |
| IIpcm1 | x | x | x | 21 | - | - | p-ti(cx) 2, p-ti(cx) 3 |
| IIpcm2 | x | x | x | 22 | - | II cpm 51 | p-cx 4, p-cx 6 |
| IIpcm3 | - | - | - | - | P.Cm | - | p-cx 5 |
| IIpcm4 | x | x | x | 36 | BA.Trm | II cpm 52 | p-tr 2 |
| IIpcm5 | - | - | - | - | P.Trm | II cpm 50 | p-tr 1 |
| IIpcm6 | x | x | x | 39 | Fm | - | s14, s16 |
| IIvlm1 | - | - | - | - | - | - | s 15 |
| IIvlm2 | - | - | - | - | iFm | - | s 13 |
| IIvlm3 | - | - | - | - | - | II vlm 38 | s 11 |
| IIvlm4 | - | - | - | - | - | - | s 12 |
| IIvlm5 | - | - | - | - | - | II vlm 39 | p-s 13 |
| IIvlm6 | x | x | - | 68 | - | - | p-s 10 |
| IIvlm7 | x | x | x | 41 | F.CmA | - | s-cx 5 |
| IIscm1 | x | x | - | - | - | II bm 57 | s-cx 3 |
| IIscm2 | - | x | - | - | - | II bm 60 | s-cx 6 |
| IIscm3 | x | x | x | 38 | F.CmP | II bm 59 | s-cx 2 |
| IIscm4 | x | - | - | - | - | II zm 61b | s-cx 4 |
| IIscm5 | - | - | - | - | - | - | s-tr1 |
| IIscm6 | x | x | x | 40 | - | II bm 58 | s-cx 1 |
| IIscm7 | - | x | - | - | - | - | s-cx 7 |
| IIscm8 | x | x | - | - | - | - | - |
| **Metathorax** | | | | | | | |
| IIIdlm1 | x | x | x | 45 | MT.m | III dlm 35 | t 14 |
| IIIdlm2 | x | x | x | 45' | S.LP?m | III dlm 36, III dlm 37 | t 12, t13 |
| IIIdlm3 | - | - | - | - | - | III dvm 40 | ? |
| IIIdvm1 | - | - | x | 46' | S.Esm | III dvm 41 | t-p 5, t-p 6 |
| IIIdvm2 | - | - | - | - | S.CmA | - | t-ti 1, t-ti 2 |
| IIIdvm3 | x | x | x | 46 | S.CmA | III dvm 43 | t-ti 3 |
| IIIdvm4 | x | x | x | 48 | - | III dvm 43 | t-cx 5 |
| IIIdvm5 | x | x | x | 49 | PSL.Cm, S.CmP | III cpm 53 | t-cx 6, t-cx 7 |
| IIIdvm6 | x | x | - | 46 | SA.Cm, SA.Fm | III dvm 42 | t-cx 8 |
| IIIdvm7 | x | - | - | - | - | III ism 44 | t-tr 1 |
| IIIdvm8 | x | x | x | 67 | - | III tpm 46a | t-s 1 |

Supplementary table 1: Homologisation of thoracic muscle nomenclatures used by several authors
"-" absent / "?" uncertain or no information

| | | | | | | | | |
|---|---|---|---|---|---|---|---|---|
| IIItpm1 | - | - | - | - | A?.Pm | III tpm 47 | t-p 3 |
| IIItpm2 | - | - | x | - | BA.Pm | III tpm 46b | t-p 4, t-p 20 |
| IIItpm3 | x | x | - | - | - | - | t-p 7, t-p 8 |
| IIItpm4 | x | x | x | 50 | SrA.Pm, Ax.Pml | - | t-p 10, t-p 11, t-p 18 |
| IIItpm5 | - | - | - | - | - | III tpm 49 | t-p 12 |
| IIItpm6 | x | x | x | 53 | - | III tpm 48 | t-p 15 |
| IIItpm7 | x | x | x | 55 | - | - | t-p 13 |
| IIItpm8 | x | x | x | 54 | - | - | - |
| IIItpm9 | x | x | x | 51/52 | Ax.PmS | III ppm 56 | t-p 14 |
| IIItpm10 | x | x | x | 56 | - | - | t-p 16 |
| IIItpm11 | - | - | - | - | - | - | t-p 19 |
| IIItpm12 | - | - | - | - | - | - | t-p 17 |
| IIItpm13 | - | - | - | - | - | ? | ? |
| IIItvm1 | x | - | - | - | - | - | - |
| IIIppm1 | - | - | - | - | - | III im 65a | p 1 |
| IIIppm2 | - | - | - | - | - | III ppm 54a, b | p 2 |
| IIIspm1 | - | - | - | - | - | III ppm 55 | p 3 |
| IIIspm2 | x | x | - | 57 | - | III zm 61 | p-s 1 |
| IIIspm3 | - | - | - | - | - | - | p-s 7 |
| IIIspm4 | - | - | - | - | - | - | p-s 9 |
| IIIspm5 | - | - | - | - | - | | p-s 5 |
| IIIspm6 | - | - | - | - | - | ? | ? |
| IIIpcm1 | x | x | x | 43 | - | - | p-ti(cx) 1 |
| IIIpcm2 | x | x | x | 44 | - | III cpm 51 | p-ti(cx) 2, p-ti(cx) 3 |
| IIIpcm3 | - | - | - | - | - | - | p-cx 4, p-cx 6 |
| IIIpcm4 | x | x | x | 58 | P.Cm | III cpm 52 | p-cx 5 |
| IIIpcm5 | - | - | - | - | BA.Trm | III cpm 50 | p-tr 2 |
| IIIpcm6 | x | x | x | 62 | P.Trm | - | p-tr 1 |
| IIIpcm7 | - | - | - | - | - | - | p-cx 8 |
| IIIvlm1 | - | - | - | - | Fm | - | s 14, s16 |
| IIIvlm2 | x | x | x | 65 | - | III vlm 64 | s 20 |
| IIIvlm3 | x | x | x | 66 | - | - | s 12 |
| - | IIIvlm4 | - | - | - | - | - | - |
| IIIscm1 | x | x | - | - | F.CmA | III bm 57 | s-cx 5 |
| IIIscm2 | x | x | - | - | - | III bm 60 | s-cx 3 |

Supplementary table 1: Homologisation of thoracic muscle nomenclatures used by several authors
"-" absent / "?" uncertain or no information

| | | | | | | | |
|---|---|---|---|---|---|---|---|
| IIIscm3 | x | x | x | 61 | - | III bm 59 | s-cx 6 |
| IIIscm4 | x | x | - | - | F.CmP | - | s-cx 2 |
| IIIscm5 | - | - | - | - | - | - | s-cx 4 |
| IIIscm6 | x | x | x | 63 | - | III cpm 58 | s-tr 1 |
| | | | | | | | |

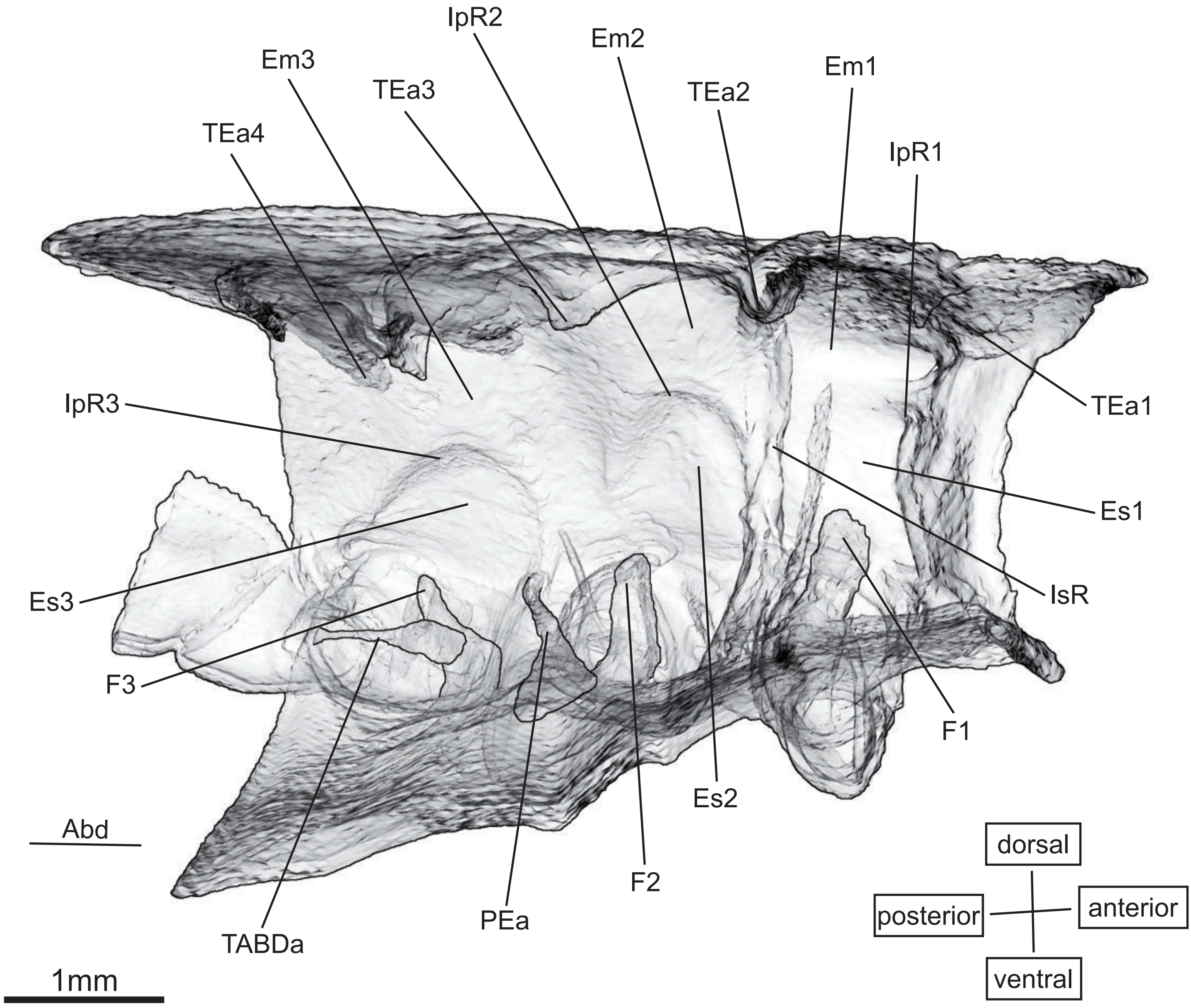


**Supplementary Figure 1:** Cuticle of *Epiophlebia laidlawi.*
3D - reconstruction from SRµCT data showing the inner pleuron of the thorax.
Abd - Abdomen, Em - epimeron, Es - episternum, F - furca, IpR - interpleural ridge, IsR - intersegmental ridge, PEa - preepisternal apodem, PEs - preepisternal sclerite, St - sternum, TABDa - thoracic-abdominal apodem, TEa - tergal apophysis.

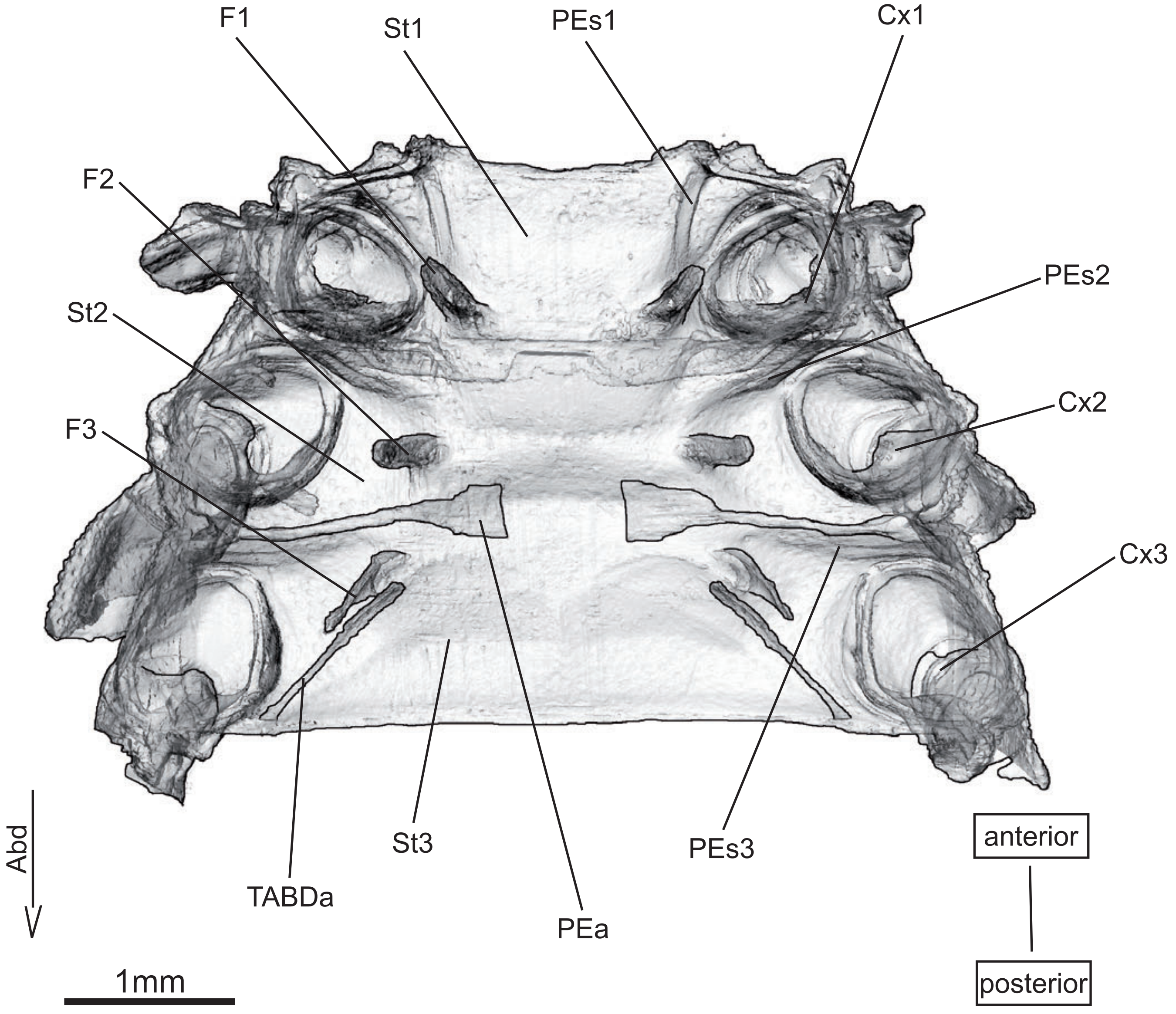


**Supplementary Figure 2**: Cuticle of *Epiophlebia laidlawi*.
3D - reconstruction from SRµCT data showing a dorsal view of the sternum of the thorax.
Abd - Abdomen, Cx - Coxa, F - furca, PEa - preepisternal apodem, PEs - preepisternal sclerite, St - sternum, TABDa - thoracic-abdominal apodem.

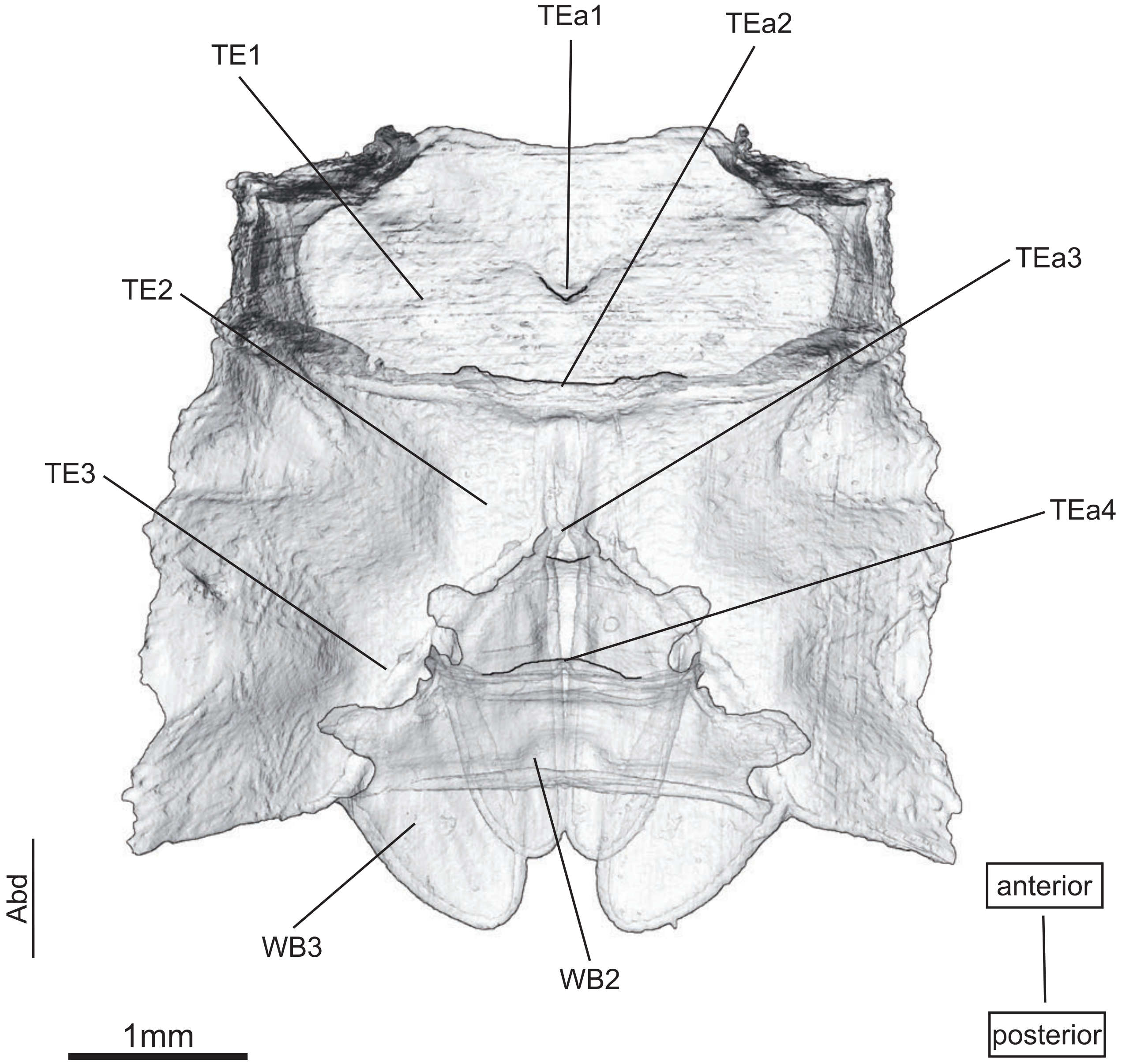


**Supplementary Figure 3:** Cuticle of *Epiophlebia laidlawi*.
3D - reconstruction from SRμCT data showing a ventral view of the tergum of the thorax.
Abd - Abdomen, TEa - tergal apophysis, TE - tergite, WB - wing bud.

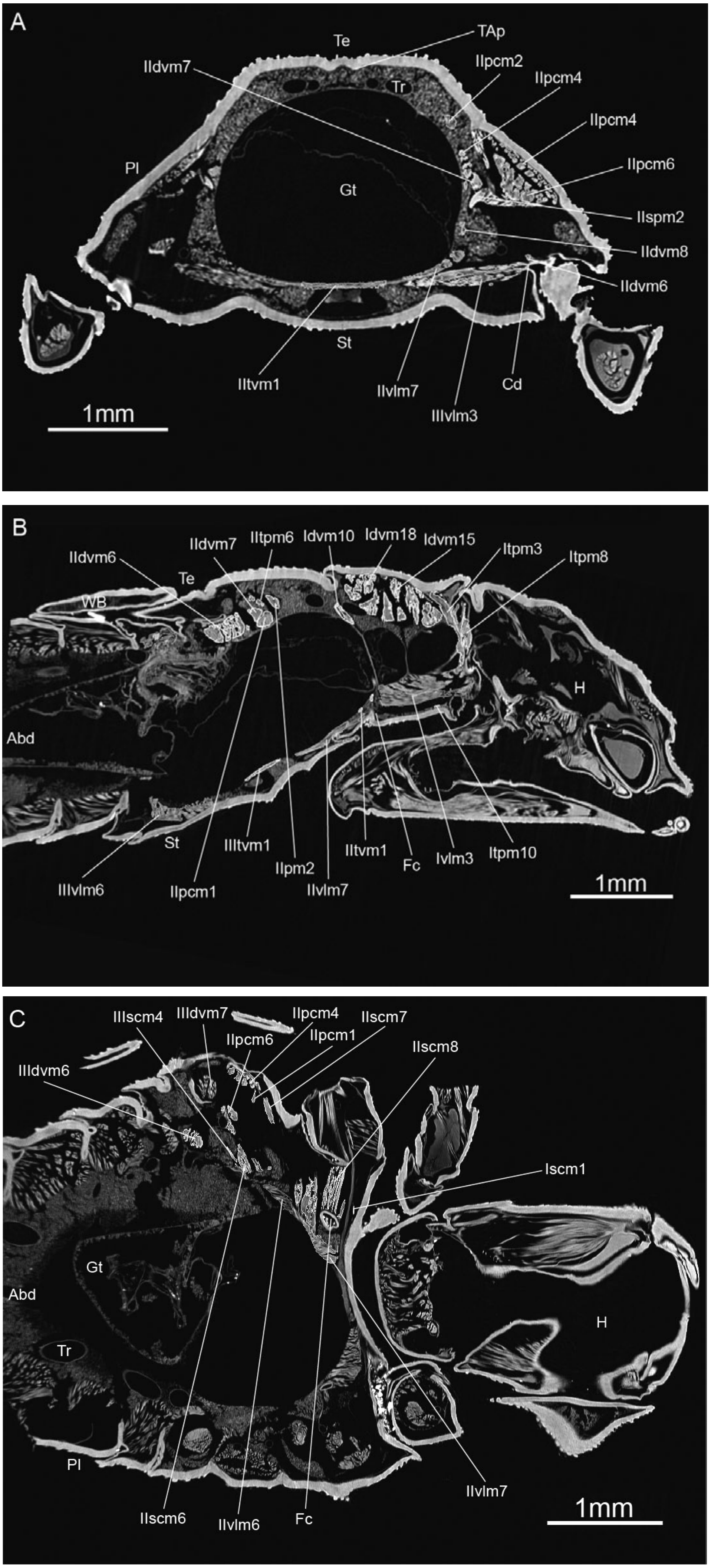


Supplementary Figure 4: Sections through head and thorax of *Epiophlebia laidlawi reconstructed from SRµCT data.* A. Cross section of the pterothorax B. Sagital section. C. Horizontal section (dorsal view)

Cd - coxal disc, dvm - dorso-vetral musculature, Gt - gut, H - head, pcm - pleuro-coxal musculature, Pl - pleura, scm - sterno-coxal musculature, St - sternum, TAp - tergal apophysis, Te - tergum, tpm - tergo-pleural musculature, Tr - trachee, vlm - ventral longitudinal musculature, WB - wing bud.

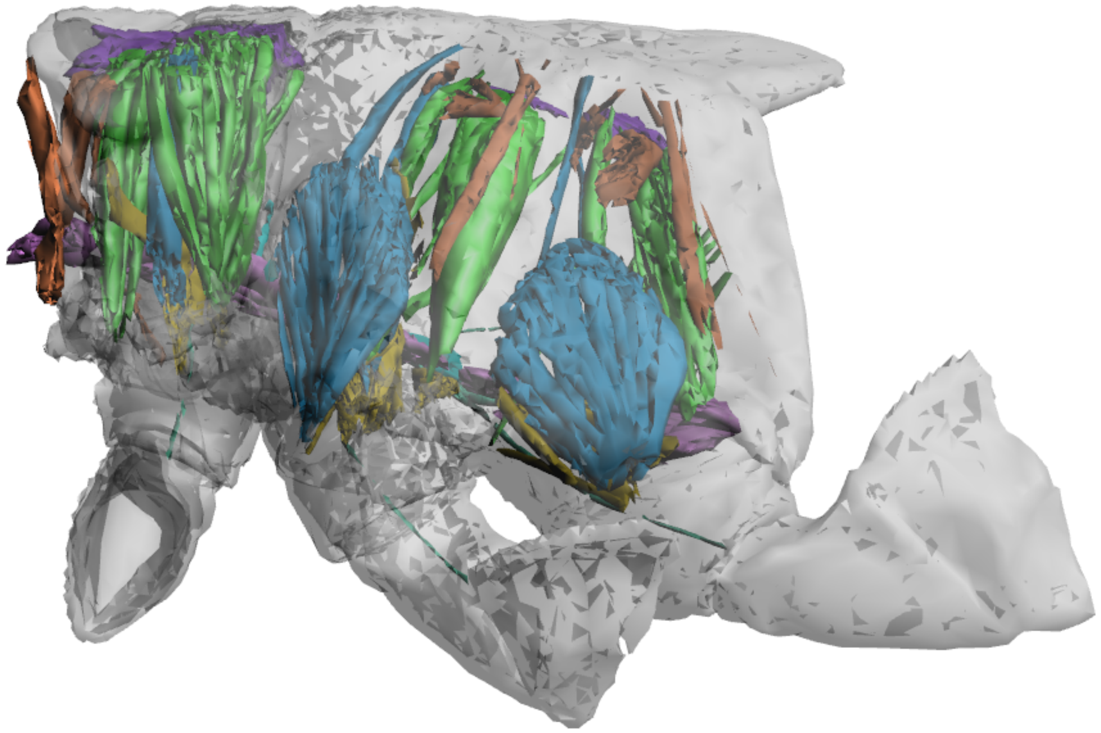

home | back | ventral | sagittal | dvm-etc | dlm-vlm

**Supplementary Figure 5:** Thoracic musculature of *Epiophlebia laidlawi*.
3D - reconstruction from SRµCT data. Click image to activate 3D, use mouse to move and manipulate virtual model.
